# Granulovacuolar degeneration bodies are neuron-selective lysosomal structures induced by intracellular tau pathology

**DOI:** 10.1007/s00401-019-02046-4

**Published:** 2019-08-27

**Authors:** Vera I. Wiersma, Anna Maria van Ziel, Sonia Vazquez-Sanchez, Anna Nölle, Ernesto Berenjeno-Correa, Anna Bonaterra-Pastra, Florence Clavaguera, Markus Tolnay, René J. P. Musters, Jan R. T. van Weering, Matthijs Verhage, Jeroen J. M. Hoozemans, Wiep Scheper

**Affiliations:** 1 Center for Neurogenomics and Cognitive Research, Department of Functional Genomics, Faculty of Science, Vrije Universiteit (VU), De Boelelaan 1085, 1081 HV Amsterdam, The Netherlands; 2 Department of Clinical Genetics, Amsterdam University Medical Centers Location VUmc, Amsterdam, The Netherlands; 3 Department of Pathology, Amsterdam University Medical Centers Location VUmc, Amsterdam, The Netherlands; 4 Department of Physiology, Amsterdam University Medical Centers Location VUmc, Amsterdam, The Netherlands; 5 Alzheimer Center, Amsterdam University Medical Centers Location VUmc, Amsterdam, The Netherlands; 6 Institut du Cerveau et de la Moelle épinière, INSERM U1127, CNRS UMR7225, Sorbonne Universités, Hôpital Pitié-Salpêtrière, Paris, France; 7 Institute of Medical Genetics and Pathology, University Hospital Basel, 4031 Basel, Switzerland

**Keywords:** Granulovacuolar degeneration bodies, Tau pathology, Casein kinase 1 δ, Lysosome

## Abstract

Granulovacuolar degeneration bodies (GVBs) are membrane-bound vacuolar structures harboring a dense core that accumulate in the brains of patients with neurodegenerative disorders, including Alzheimer’s disease and other tauopathies. Insight into the origin of GVBs and their connection to tau pathology has been limited by the lack of suitable experimental models for GVB formation. Here, we used confocal, automated, super-resolution and electron microscopy to demonstrate that the seeding of tau pathology triggers the formation of GVBs in different mouse models in vivo and in primary mouse neurons in vitro. Seeding-induced intracellular tau aggregation, but not seed exposure alone, causes GVB formation in cultured neurons, but not in astrocytes. The extent of tau pathology strongly correlates with the GVB load. Tau-induced GVBs are immunoreactive for the established GVB markers CK1δ, CK1ɛ, CHMP2B, pPERK, peIF2α and pIRE1α and contain a LAMP1- and LIMP2-positive single membrane that surrounds the dense core and vacuole. The proteolysis reporter DQ-BSA is detected in the majority of GVBs, demonstrating that GVBs contain degraded endocytic cargo. GFP-tagged CK1δ accumulates in the GVB core, whereas GFP-tagged tau or GFP alone does not, indicating selective targeting of cytosolic proteins to GVBs. Taken together, we established the first in vitro model for GVB formation by seeding tau pathology in primary neurons. The tau-induced GVBs have the marker signature and morphological characteristics of GVBs in the human brain. We show that GVBs are lysosomal structures distinguished by the accumulation of a characteristic subset of proteins in a dense core.

**Electronic supplementary material:**

The online version of this article (10.1007/s00401-019-02046-4) contains supplementary material, which is available to authorized users.

## Introduction

The intracellular deposition of aggregates composed of the microtubule-binding protein tau is the characteristic neuropathological hallmark of a group of neurodegenerative diseases that are collectively called tauopathies, including Alzheimer’s disease (AD) [62]. In the brains of tauopathy patients, tau pathology is commonly accompanied by another neurodegenerative change: granulovacuolar degeneration (GVD) [37]. GVD is characterized by the presence of membrane-delineated vacuoles harboring a dense core—the so-called GVD bodies (GVBs)—predominantly in the neuronal soma [68]. Although the phenomenon has first been reported more than a century ago, the etiology of GVD and the identity of GVBs are still largely elusive [61]. Immunohistochemical analyses of post-mortem brain indicate the presence of a myriad of proteins in GVB cores, including proteins of the unfolded protein response (UPR) [30, 50], several other cellular stress-related proteins [39, 81] and cytoskeletal components [15] (for review see Ref. [37]). In addition, the core and membrane of GVBs contain prototypical markers of the autophagic and endocytic pathways, which deliver intracellular and extracellular cargo, respectively, to the lysosome for degradation [20]. The most commonly used GVB markers that consistently detect GVD in human brain tissue include the endosomal protein charged multivesicular body protein 2b (CHMP2B), the UPR activation markers phosphorylated protein kinase R (PKR)-like endoplasmic reticulum kinase (pPERK), eukaryotic translation initiation factor 2 α (peIF2α) and inositol-requiring enzyme 1 α (pIRE1α) and the casein kinase 1 (CK1) isoforms δ and ɛ [23, 30, 78].

In AD patients the amount of neurons carrying GVBs is significantly increased compared to age-matched controls [3, 76]. Also in the brains of patients with primary tauopathies, including Pick’s disease, progressive supranuclear palsy (PSP) and frontotemporal dementia (FTD) caused by *MAPT* mutations, the GVB abundance is significantly higher than in elderly controls [50, 64, 76]. Moreover, GVBs have been reported in various other disorders hallmarked by tau pathology, including corticobasal degeneration (CBD) [39, 67], argyrophilic grain disease [67], pallido-ponto-nigral degeneration [60], parkinsonism dementia complex of Guam [60], Down syndrome [9, 60], pantothenate kinase-associated neurodegeneration [16, 77] and post-fetal cases of Fukuyama-type congenital muscular dystrophy [58]. This wide spectrum of tauopathies includes primary and secondary as well as sporadic and familial variants, demonstrating that independent of disease etiology, tau pathology and GVBs coincide.

In the AD hippocampus the number of neurons with GVBs increases with the Braak stage for neurofibrillary tau tangles (NFTs) and thus strongly correlates with the local tau pathology load [6, 22, 30, 42]. In addition, the distribution pattern of GVBs through the AD brain, as described by the Thal staging, follows the stereotypical spreading pattern of tau pathology [67]. Accordingly, the GVB load correlates with the severity of cognitive symptoms in AD [22, 35]. At the cellular level, GVBs are typically detected in neurons with tau pathology. More specifically, GVBs predominate in neurons with early rather than end-stage tau pathology in AD and FTD subtypes [30, 37, 50, 77], suggesting a potential involvement in the pathogenesis.

Tau pathology is commonly modeled in transgenic (Tg) animal models by overexpression of human tau containing FTD-causing mutations. GVBs have been detected in the brains of tau P301L Tg mice, but not in Tg mouse models for beta-amyloid (Aβ) amyloidosis or non-Tg animals [33, 38, 41, 45]. However, in these tau Tg models GVBs are rare, which has limited further insight into their origin and function. In the last decade novel animal models of tauopathies have been developed that employ injection of fibrillar tau assemblies—so-called “seeds”—into the brains of tau Tg mice, resulting in accelerated induction of tau pathology [13, 31, 52].

In the present study, we aimed to establish experimental models for GVB formation, in order to gain more insight into the identity of GVBs. We show that seeding-induced intracellular tau pathology triggers GVB formation in vivo and report the first in vitro model for GVB formation by seeding tau pathology in cultured primary neurons. We employed the neuronal in vitro assay for a detailed characterization of GVBs and demonstrate that GVBs are proteolytically active lysosomal compartments that accumulate endocytic as well as specific cytosolic cargo.

## Materials and methods

### In vivo seeding with recombinant PFFs

Murine material to study in vivo seeding of tau pathology by injection of recombinant preformed fibrils (PFFs) came from a study previously performed at Janssen Pharmaceutica (Beerse, Belgium) according to local legislation. The experimental procedures have been described elsewhere [52]. Briefly, synthetic human K18 tau P301L PFFs were generated using heparin and separated from the fibrillization mixture by ultracentrifugation at 100,000*g* for 1 h, followed by washing, resuspension of the pellet in ammonium acetate buffer (0.1 M, pH 7.0) and sonication. The K18 domain spans residues 244–372 of the longest human tau isoform and includes the four microtubule-binding repeat domains. For the injection of PFFs into mice, 3-month-old homozygous human tau P301L Tg mice on a FVB/N background [66] (*N *= 5, 3 females, 2 males) were deeply anesthetized with isoflurane. The hippocampus (AP − 2.0 mm, ML + 2.0 mm from bregma; DV − 2.0 from dura) received a unilateral (right hemisphere) stereotaxic injection of 25 µg sonicated PFFs (injection volume 5 µL) at a speed of 1 µL/min. Following injection, the needle was kept in place for an additional 5 min before gentle withdrawal. As a control, tau P301L Tg mice (*N *= 5, 2 females, 3 males) were injected with ammonium acetate buffer following the same injection procedure. Mice were sacrificed 2 months post-injection, a time point prior to the onset of tau pathology as a result of transgene expression.

### In vivo seeding with mouse and human brain homogenates

Murine material to study in vivo seeding of tau pathology by the injection of human and mouse brain lysates came from studies performed and described previously [12, 13]. Homozygous wild-type (WT) human tau Tg ALZ17 mice (a strain in which transgene expression does not lead to filamentous tau pathology) [55], homozygous human tau P301S Tg mice (a strain in which transgene expression results in the development of filamentous tau aggregates) [1] and non-Tg control mice, all females on a C57BL/6 background, were used. Experiments were performed in compliance with protocols approved by the local Basel Committee for Animal Care and Animal Use.

Mouse brain extract was prepared by combining three brainstems of 6-month-old tau P301S Tg mice or non-Tg C57BL/6 mice control mice. Human brain material for homogenization was derived from neuropathologically confirmed cases of AD, PSP and tangle-only dementia (TD) and an age-matched control. Patient details and brain regions used for experiments are listed in Supplementary Table 1 in Online Resource 1. Informed consent for research purposes is available for all samples. Mouse and human brain tissue was homogenized in phosphate-buffered saline (PBS) at 10% (wt/vol) for murine tissues and 20% (wt/vol) for human tissues, briefly sonicated (Branson 450, output 2, 5 times 0.9 s) and centrifuged at 3000*g* at 4 °C for 5 min. The supernatant was aliquoted, snap-frozen and kept at − 80 °C until use. 5 µL of 40 times diluted brain homogenate was analyzed on Western blot (performed as previously described [13]) to show the presence of tau and phospho-tau. Analyses of the mouse brain extracts used in this study have been published previously [13].

For the immunodepletion of tau, a mixture of the anti-tau antibodies HT7 (Pierce), AT8 (Pierce) and tau-5 (Biosource) (20 µg of each antibody) was incubated for 2 h with 10 µL of Protein G Sepharose 4 Fast flow (PGS, Pharmacia). Sepharose beads were washed twice with 1 mL PBS and the liquid removed with a glass capillary. Tau P301S Tg mouse brain extract (100 µL, prepared as described above) was added to semi-dry beads and allowed to react overnight at 4 °C on an overhead mixer. As a control, tau P301S Tg mouse brain extract was treated as described above, but omitting the anti-tau antibodies. The beads were sedimented by centrifugation (200*g*, 1 min) and 70 µL of the supernatant was removed and stored at − 80 °C until use. Western blot analysis confirming tau depletion in the material used in this study has been published before [13].

For the injection of brain extract into mice, 3-month-old ALZ17 mice were anesthetized with a mixture of ketamine (10 mg/kg) and xylazine (20 mg/kg). When deeply anesthetized, the mice were placed on a heating pad to maintain body temperature during surgery. Using a Hamilton syringe, the hippocampus (A/P, − 2.5 mm from bregma; L, ± 2.0 mm; D/V, − 1.8 mm) and the overlying cerebral cortex (A/P, − 2.5 mm from bregma; L, ± 2.0 mm; D/V, − 0.8 mm) each received a unilateral (right hemisphere) stereotaxic injection of 2.5 µL brain extract, at a speed of 1.25 µL/min. Following injection, the needle was kept in place for an additional 3 min before gentle withdrawal.

Mice injected with brain lysate derived from tau P301S Tg mice (*N *= 6) were sacrificed 12 (1 mouse), 15 (4 mice) and 17 (1 mouse) months post-injection. As controls, ALZ17 mice were injected with non-Tg C57BL/6 mouse brain extract (*N *= 1) and sacrificed 19 months after injection or with tau P301S Tg mouse brain extract immunodepleted of tau (*N* = 4) and sacrificed 6 (1 mouse) or 12 (3 mice) months post-injection. ALZ17 mice receiving human tauopathy [AD (*N *= 6), PSP (*N *= 3), TD (*N *= 4)] or human control (*N *= 4) brain extract were sacrificed 6 (AD: 2 mice, TD: 1 mouse, PSP: 2 mice), 14 (Ctrl: 1 mouse) or 15 (AD: 4 mice, TD: 3 mice, PSP: 1 mouse, Ctrl: 3 mice) months post-injection.

### Immunolabeling of mouse and human brain tissue

Formalin-fixed, paraffin-embedded sagittal (recombinant tau seeding model) or coronal (brain-derived tau seeding model) hippocampal sections of injected mice were analyzed. For reference, formalin-fixed, paraffin-embedded hippocampal sections of confirmed AD patients (see Supplementary Table 1 in Online Resource 1 for patient details) were used (Netherlands Brain Bank, informed consent for research purposes available).

For immunofluorescence, the same protocol was used for mouse and human brain sections with exception of the mouse-to-mouse blocking procedure on mouse sections and Sudan Black on human sections. Sections were deparaffinized and antigen-retrieval was performed by heat-treatment in sodium citrate buffer (0.01 M, pH 6.0). Mouse tissue sections were incubated with mouse-to-mouse blocking solution (CleanVision, Immunologic) for 30 min at room temperature (RT) prior to primary antibody incubation. Sections were incubated with a mixture of primary antibodies diluted in antibody diluent (Sigma-Aldrich) overnight (o/n) at RT. Primary antibodies and dilutions are listed in Table 1. Staining controls consisting of omission of one or both primary antibodies were performed for all stainings. Sections were incubated with Alexa Fluor 488-conjugated goat-anti-mouse and Alexa Fluor 555-conjugated goat-anti-rabbit secondary antibodies (Invitrogen) diluted in antibody diluent for 2.5 h at RT (secondary antibody concentration 1:400) or for 1 h at RT (secondary antibody concentration 1:250). To visualize nuclei, sections were incubated with 4′,6-diamidino-2-phenylindole (DAPI, Invitrogen) diluted in PBS (6.67 µg/mL) for 10 min at RT. Human tissue sections were additionally incubated with Sudan Black (0.2% in 70% ethanol, Sigma-Aldrich) for 10 min at RT to quench autofluorescence. Between incubation steps, sections were rinsed with PBS. Sections were mounted in 80% glycerol in Tris-buffered saline (TBS) and stored at 4 °C until imaging.

**Table 1:** Overview of the primary antibodies used in the present study

Antibody	Phospho-epitope	Species	Dilution						Source	Product number
			Tissue		In vitro					
			IF	IHC	Confocal	STED	TEM	WB		
Acetylated α-tubulin		Mouse monoclonal			1:100				Sigma-Aldrich	T7451
AT100	Thr212/Ser214	Mouse monoclonal	1:1000		1:200				ThermoScientific	MN1060
AT8	Ser202/Thr205	Mouse monoclonal	1:800	1:400	1:400			1:500/1:1000	ThermoScientific	MN1020
β-III-tubulin		Mouse monoclonal			1:500				Merck Millipore	MAB1637
Calnexin		Rabbit polyclonal			1:250				Enzo Life Sciences	ADI-SPA-860
CHMP2B [4]		Rabbit polyclonal			1:200			1:1000	Abcam	ab33174
CK1δ		Mouse monoclonal			1:1000	1:100	1:100	1:1000	Santa Cruz Biotechnology	sc-55553
CK1δ		Rabbit polyclonal	1:400		1:500				ThermoScientific	PA5-32129
CK1ɛ		Rabbit polyclonal			1:100				Santa Cruz Biotechnology	sc-25423
c-Myc		Mouse monoclonal			1:50				Santa Cruz Biotechnology	sc-40
c-Myc		Chicken polyclonal			1:50				Aves Labs	ET-MY100
CTSD		Rabbit polyclonal			1:100				Merck Millipore	06-467
EEA1		Rabbit polyclonal			1:100				Cell Signaling	2411
GAPDH		Mouse monoclonal						1:2500	Merck Millipore	MAB374
GFAP		Mouse monoclonal			1:1000				Sigma-Aldrich	G3893
GRASP55		Rabbit polyclonal			1:100				Atlas Antibodies	HPA035275
G3BP		Mouse monoclonal			1:100				BD Transduction Laboratories	611126
HT7		Mouse monoclonal						1:1000	ThermoScientific	MN1000
LAMP1		Rat monoclonal			1:100				Abcam	ab25245
LC3 [34, 46]		Mouse monoclonal			1:50				MBL	M152-3
LIMP2/lpg85		Rabbit polyclonal			1:500	1:100			Novus Biologicals	NB400-129
MAP2		Chicken polyclonal			1:500				Abcam	ab5392
MC1		Mouse monoclonal			1:500				Kind gift from Dr. Peter Davies	
peIF2α	Ser51	Rabbit polyclonal	1:100						Sigma-Aldrich	E2152
peIF2α	Ser51	Rabbit polyclonal			1:50				Cell Signaling	9721
pIRE1α	Ser724	Rabbit polyclonal	1:1250		1:50				Novus Biologicals	NB100-2323
pPERK	Thr981	Rabbit polyclonal	1:6000	1:800	1:1000				Santa Cruz Biotechnology	sc-32577
Tau-5		Mouse monoclonal						1:1000	Abcam	ab80579
VGAT		Rabbit polyclonal			1:500				Synaptic Systems	131002
VGLUT1		Guinea pig polyclonal			1:1000				Merck Millipore	AB5905

Target protein and species the primary antibody was raised are listed. For phospho-specific antibodies the phospho-epitope is indicated. The dilution used in different experimental approaches, the source and product number are listed. For some antibodies, references to studies validating the antibody are included

*IF* immunofluorescence, *IHC* immunohistochemistry, *STED* stimulated emission depletion (microscopy), *TEM* transmission electron microscopy, *WB* Western blotting

For immunohistochemistry, the same protocol was used for mouse and human brain sections. After deparaffinization, endogenous peroxidase activity was blocked in 0.3% H_2_O_2_ in methanol (MeOH) for 30 min. Sections were incubated with the primary antibody pPERK (Table 1) diluted in antibody diluent o/n at 4 °C. Subsequently, sections were incubated with horseradish peroxidase (HRP)-labeled goat-anti-mouse/rabbit secondary antibody (EnVision detection system, DAKO) for 60 min at RT and developed using the chromogen 3′-3′-diaminobenzidine (DAB, DAKO). Hereafter, antigen-retrieval was performed by heat-treatment in sodium citrate buffer (0.01 M, pH 6.0), sections were blocked with normal rabbit serum (DAKO) (1:50 in antibody diluent) for 10 min, followed by incubation with AT8 (Table 1) diluted in antibody diluent for 1 h at RT. Staining controls consisting of omission of one or both primary antibodies were performed. Sections were incubated with biotinylated rabbit-anti-mouse secondary antibody (DAKO) (1:500 in antibody diluent) for 30 min at RT, incubated with alkaline phosphatase-conjugated streptavidin (DAKO) (1:1000 in antibody diluent) for 60 min at RT, washed with TBS and subsequently developed using the chromogen Liquid Permanent Red (LPR, DAKO). Nuclei were counterstained with haematoxylin. Between incubation steps, sections were rinsed with PBS and/or tap water. Sections were dehydrated and mounted using the non-aqueous mounting medium VectaMount (Vector Laboratories).

### Analysis of immunofluorescence labeling of mouse tissue

Z-stacks (15 steps of 1 µm) were acquired using a Nikon Eclipse Ti confocal microscope equipped with a 60 × oil immersion objective (NA = 1.4) controlled by NisElements 4.30 software. For quantification of the number of immunoreactive cells in the recombinant tau seeding model, images were acquired using a Leica DMi8 inverted fluorescence microscope equipped with a Leica DFC300 G camera and 40 × objective controlled by Leica Application Suite (LAS) 1.5 software. Non-overlapping images were taken covering the CA1, CA2 and CA3 area of the hippocampus. Images were analyzed using LAS Advanced Fluorescence 2.6.3 software. Pyramidal neuron nuclei were counted in the DAPI channel. The number of neurons with AT8-positive tau pathology or CK1δ-labeled GVBs was counted separately. Subsequently, the number of double-positive neurons was counted. Percentages of single and double-positive cells were calculated per mouse (see Supplementary Table 2 in Online Resource 1 for an overview of the number of cells analyzed). For the analysis of GVB core and neuronal soma area in human and PFF-injected mouse hippocampus, see below (“Confocal microscopy image analysis” section).

### Animals and (primary) cell culture

Animal experiments for primary cell culture are in accordance with institutional and Dutch governmental guidelines and regulations and were approved by the animal ethical committee of the VU University/VU University Medical Center.

Embryonic day (E) 18.5 WT mouse embryos were obtained by cesarean section of pregnant females from timed mating and used for primary cultures. For neuronal cultures, hippocampi, cortices and striata were dissected in Hanks buffered salt solutions (HBSS, Sigma-Aldrich) containing 10 mM HEPES (Gibco) (Hanks–HEPES) and digested by addition of 0.25% trypsin (Gibco) for 20 (hippocampi and cortices) or 30 (striata) minutes at 37 °C. Hereafter, digested tissue was washed three times in Hanks–HEPES and subsequently triturated with fire-polished Pasteur pipettes in DMEM containing 4.5 g/L glucose and UltraGlutamine I (DMEM, Lonza) supplemented with 10% heat inactivated fetal bovine serum (HI-FBS), 1% penicillin/streptomycin (pen-strep) and 1% non-essential amino acid solution (Gibco) (DMEM +). Dissociated cells were spun down, resuspended and plated in Neurobasal medium (Gibco) supplemented with 2% B-27 (Gibco), 18 mM HEPES, 0.25% Glutamax (Gibco) and 0.1% pen-strep (NB +).

For Western blotting, cortical neurons were plated in 6-well plates (600,000 or 700,000 neurons/well). For immunolabeling, neurons were plated on 13 mm glass coverslips (ThermoFisher Scientific) in 24-well plates (40,000 hippocampal, 40,000–60,000 cortical, 80,000 striatal neurons/well). For analysis using an automated screening platform, neurons were plated in 96-well plates (15,000 hippocampal or cortical neurons/well). Unless otherwise stated, hippocampal neurons were used for immunolabeling experiments.

Cortices from E18.5 mouse embryos were used for astrocytic cultures. Cortices were digested using papain (Worthington Biochemical Corporation, 20–25 U/mL) in DMEM containing 4.5 g/L glucose and UltraGlutamine I (DMEM, Lonza) supplemented with 0.2 mg/mL l-cysteine (Sigma), 1 mM CaCl2 (Sigma) and 0.5 mM EDTA (AppliChem) for 45 min at 37 °C. Hereafter, cortices were incubated for 15 min at 37 °C with inactivating solution consisting of DMEM containing 4.5 g/L glucose and UltraGlutamine I (DMEM, Lonza) supplemented with 10% HI-FBS, 2.5 mg/mL albumin (Applichem) and 2.5 mg/mL trypsin inhibitor type II-O (Sigma). Cells were dissociated by trituration with fire-polished Pasteur pipettes and cultured in DMEM + in T75 culture flasks till confluency. Astrocytes were then re-plated into wells plates. Upon reaching 80% confluency the medium was changed to NB +. For Western blotting, astrocytes were plated in 6-well plates (300,000 astrocytes/well). For immunofluorescence, astrocytes were plated on 13 mm glass coverslips in 24-well plates (50,000 astrocytes/well).

The human embryonic kidney 293 (HEK293) stable cell line with inducible expression of human 2N4R tau P301L coupled to green fluorescent protein (GFP) has been described previously [10]. HEK293 cells were maintained in T75 culture flasks in DMEM supplemented with 10% HI-FBS and 1% Pen-Strep. Cells were regularly treated with 200 µg/mL Zeocin (Invitrogen). For Western blotting, HEK293 cells were plated in 6-well plates (80,000 or 300,000 cells/well). For immunofluorescence, HEK293 cells were plated on 13 mm glass coverslips in 24-well plates (10,000 cells/well). 24 h after plating the medium was changed to medium containing 100 ng/mL doxycycline (Sigma-Aldrich) to induce expression of the tau transgene.

For all cell types, plates and glass coverslips were coated with a 5 µg/mL poly-l-ornithine (Sigma) and 2.5 µg/mL laminin (Sigma) coating solution o/n at RT. Cells were maintained at 37 °C, 5% CO_2_.

### Lentiviral constructs

Lentiviral transductions were performed at 3 days in vitro (DIV) or 3 days after medium change to NB + for primary mouse neurons and astrocytes, respectively.

The following 2N4R human tau constructs were used: tau P301L without a fluorescent tag (tau-P301L), tau P301L containing an in-frame C-terminal EGFP tag (tau-P301L-GFP) or tau P301L containing an in-frame C-terminal mCherry tag (tau-P301L-mCherry) in a pLenti second-generation lentiviral backbone vector under the cytomegalovirus (CMV) promoter.

WT mouse CK1δ (kind gift from Dr. Y.E. Greer) has been described [25]. To generate the CK1δ-EGFP fusion protein the construct was cloned by seamless ligation independent cloning into the entry vector pEGFP-C1 (Clontech). The resulting construct was subcloned into a pLenti vector under the CMV promotor using the Gateway system. A CMV-driven pLenti EGFP construct was used as a control. Lentiviral particles were generated as described previously [48].

### Tau seeding

For in vitro fibril production, 40 µM recombinant K18 tau P301L was incubated with 10 µM low-molecular-weight heparin and 2 mM DTT in sodium acetate buffer (0.1 M, pH 7.0) for 24 h at 37 °C. PFFs were diluted in sodium acetate buffer, sonicated using a probe sonicator (25 pulses with 3 s rest every 5th pulse) and stored at − 80 °C until use. Unless otherwise stated, PFFs were added directly to the culture medium. Primary neurons and astrocytes were treated with PFFs at DIV 7 and 7 days after medium change to NB +, respectively. PFF concentrations between 20 and 200 nM were used. HEK293 cells were treated with 200 nM PFFs 24 h after doxycycline treatment. Equal volumes of sodium acetate buffer were added to control cultures.

For the direct intracellular delivery of PFFs, Lipofectamine-2000 (Invitrogen) was used. Lipofectamine-2000 was incubated with PFFs or an equal volume of sodium acetate buffer diluted in Optimem (Gibco) for 20 min at RT. Before addition of the lipofectamine mixture, half of the culture medium was refreshed and stored at 4 °C (conditioned medium). Lipofectamine mixture was added drop-wise to the culture medium and incubated for 3–4 h at 37 °C followed by medium change to pre-warmed conditioned medium plus fresh medium.

### Medium refreshment

Fresh medium (40% of the total volume in the well) was supplied to the cultures at DIV 10 for neurons, at 10 days after medium change to NB + for astrocytes and 4 days after PFF treatment for HEK293 cells.

### Cell treatments

For visualization of proteolytically active compartments, neurons at DIV 17 were incubated with 10 µg/mL DQ-BSA Red (Invitrogen) diluted in PBS for 18 h at 37 °C. An equal volume of PBS was added to control cultures. As positive control for LC3 and G3BP stainings, neurons were treated with 30 nM bafilomycin A1 (Sigma-Aldrich) for 1 h or 10 µM thapsigargin (Santa Cruz) for 24 h at 37 °C, respectively. To disrupt microtubules, neurons at DIV 14 were treated with 25 µM vinblastine (Tebu-bio) for 24 h at 37 °C. Equal volumes of dimethyl sulfoxide (DMSO) were included as vehicle control.

### Fixation

Neurons and astrocytes were fixed 11 days after PFF treatment at DIV 18 and 18 days after medium change to NB +, respectively. HEK293 cells were fixed 7 days after PFF treatment. Cells were fixed in 1.85% formaldehyde (FA, Electron Microscopy Sciences) in PBS (pH 7.4) by addition to the culture medium for 10 min followed by fixation in 3.7% FA for 10 min at RT. In experiments in which DQ-BSA Red was used, cells were washed two times with PBS prior to fixation in 3.7% FA for 15 min at RT. To remove soluble tau, cells were fixed in ice-cold MeOH for 15 min at − 20 °C while shaking. For stimulated emission depletion (STED) imaging, neurons were fixed in 4% paraformaldehyde (PFA, Merck) in PBS (pH 7.4) for 10 min at RT. For electron microscopy, neurons were fixed in 2% PFA by addition to the culture medium for 10 min followed by fixation in 4% PFA for 30 min at RT. After fixation, cells were washed with PBS and stored at 4 °C.

### Immunofluorescence followed by confocal or automated microscopy

Fixed cells on coverslips or cells in 96-well plates were permeabilized in 0.5% Triton X-100 (ThermoFisher Scientific) in PBS (pH 7.4) for 5 min at RT and blocked in 2% goat serum (Gibco) and 0.1% Triton X-100 in PBS for 30 min at RT. Cells were incubated with primary antibodies diluted in blocking solution o/n at 4 °C, with exception of the microtubule-associated protein 2 (MAP2) antibody which was also used for 2 h at RT. Primary antibodies and dilutions are listed in Table 1. After washing in PBS, cells were incubated with Alexa Fluor (405, 488, 546, 647)-conjugated secondary antibodies (Invitrogen and Abcam) diluted in blocking solution (1:500) for 1 h at RT. Antibody incubations were performed sequentially. Staining controls consisting of omission of primary antibodies were included. Following PBS washes, nuclei were stained with DAPI (Brunschwig Chemie) diluted in PBS (5 µg/mL).

Coverslips were mounted on microscope slides (ThermoFisher Scientific) using Mowiol (Sigma-Aldrich). Cells were imaged using a Nikon Eclipse Ti confocal microscope equipped with a 60 × oil immersion objective (NA = 1.4) controlled by NisElements 4.30 software (Nikon). Single focal planes or Z-stacks with a step size of 0.3 or 1 µm were obtained. Unless otherwise stated, maximum intensity projected Z-stacks are shown. The number of independent experiments and number of cells analyzed are indicated in the figure legend or in Supplementary Table 3 in Online Resource 1.

Single focal plane images from neurons in 96-well plates were acquired using the 20 × objective a CellInsight CX7 High-Content Screening (HCS) Platform (ThermoFisher Scientific) controlled by HCS Studio Cell Analysis software (ThermoFisher Scientific). 100 images were acquired per well to cover the majority of the well surface. The DAPI channel was used for autofocus.

### Confocal microscopy image analysis

Unless otherwise stated, single focal planes were analyzed using ImageJ software (National Institutes of Health). Observations from independent experiments were pooled for analysis.

Line segments were drawn to determine the intensity values plotted in the form of line graphs which show fluorescence intensity in arbitrary units (AU).

For the analysis of GVB core area in human and PFF-seeded mouse hippocampus and PFF-seeded primary hippocampal mouse neurons, a threshold was applied in the CK1δ channel, neighboring GVBs were separated by watershed segmentation and particle area in µm^2^ was quantified. The soma area of GVB-containing neurons in human and mouse hippocampus was quantified by measuring the area in µm^2^ of a region of interest (ROI) drawn using the AT8 signal of maximum intensity projected Z-stacks.

For the analysis of the fluorescence AT100 intensity in neurons, images were acquired using the MAP2 channel for autofocus. In the control buffer-treated cultures, cells were randomly sampled. In the PFF-treated condition, cells with GVBs were manually selected based on the presence of CK1δ-labeled GVBs. Subsequently, cells without GVBs were manually selected in the CK1δ channel. Both groups were selected blinded for the AT100 signal. The intensity of the AT100 signal was determined within a MAP2-based ROI. Non-specific nuclear AT100 signal [74] was removed from the analysis using a DAPI mask. Values were normalized to the control condition, which was set to 1.

DQ-BSA fluorescence intensity was determined for CK1δ-positive (GVBs) and -negative (non-GVBs) LIMP2-positive structures by defining ROIs in the LIMP2 channel. The average DQ-BSA intensity of four ROIs outside LIMP2-positive structures within the MAP2-delineated neuronal cytosol was defined as background per cell. Multiple focal planes per Z-stack were analyzed. DQ-BSA intensities in LIMP2-positive structures are expressed as signal/background intensity ratio.

For the quantification of the accumulation of overexpressed CK1δ-GFP and GFP in GVBs, fluorescence intensity was determined in a ROI defined by applying a threshold in the CK1ɛ channel. A background ROI was defined in the neuronal cytosol outside GVBs by applying a threshold in the MAP2 channel excluding the GVB ROIs. Values are expressed as GVB/cytosol fluorescence intensity ratio.

### Automated microscopy image analysis

Images obtained by automated microscopy were analyzed by in-house developed scripts using Columbus 2.5 software (PerkinElmer). Mean nuclei number per well was determined based on the DAPI signal and single well observation values were used for analysis. The pathological tau signal (AT100 or MeOH-insoluble tau-P301L-GFP fluorescence intensity) was determined within a MAP2-based ROI. To measure GVB load, images were pre-processed to remove smooth and continuous background intensity from the CK1δ or pPERK signal. Bright punctate signal was selected based on a set of criteria, including intensity and morphological properties. Puncta within a specified distance were clustered and counted as one object thus determining the number of neurons containing GVBs. For both the tau pathology and GVB read-outs, background measured in the control condition was subtracted from all conditions. For the analysis of tau pathology and GVB load in cells with and without tau-P301L expression and PFF treatment, single well observation values were used for analysis. Per experiment, values were normalized to the control condition. The two observations with the highest tau or GVB load detected were set to 100 and all values re-scaled accordingly. For the analysis of the seed dose–response, per experiment the lowest tau and GVB load observed across all conditions were set to 0% and the highest to 100%. Single observations were averaged per experiment and used for analysis. For the analysis of tau pathology and GVB load in hippocampal and cortical cultures, background subtraction was performed using control buffer-treated cultures from the same neuronal subtype and the ratio GVB load/tau pathology load was calculated per well. The average ratio in hippocampal neurons was arbitrarily set to 1.

### Immunofluorescence followed by STED imaging

For STED imaging experiments, neurons were not permeabilized after fixation, but directly blocked in PBS supplemented with 5% normal goat serum for 1 h at RT. Neurons were simultaneously incubated with the primary antibodies CK1δ and LIMP2 diluted in 1% bovine serum albumin (BSA) in PBS (1:100) o/n at 4 °C (Table 1). Following washes with PBS, cells were incubated with STAR 580- and STAR 635P-conjugated secondary antibodies (Abberior) diluted in 1% BSA in PBS (1:50) o/n at 4 °C. After PBS washes, nuclei were stained with DAPI diluted in PBS (5 µg/mL). Coverslips were mounted on microscope slides in Mowiol containing 2.5% Dabco (Sigma-Aldrich).

STED was performed on a Leica TCS SP8 STED 3 × microscope (Leica Microsystems) with LAS X acquisition software using a dedicated 100 × oil objective (NA = 1.4). Samples were excited with a pulsed white light laser at maximum excitation efficiency. The signal was detected using a gated hybrid detector (HyD) (Leica Microsystems) in photon-counting mode. The pinhole was set to 0.71 Airy units at 580 nm emission, using the “smart pinhole” function in LAS X. A continuous wave STED laser at a wavelength of 592 nm was used for depletion of the tau-P301L-GFP signal reaching a lateral resolution of ~ 155 nm. A pulsed STED laser line at a wavelength of 775 nm was used for the depletion of the 580 and 635 nm fluorophores, reaching a lateral resolution of ~ 155 and ~ 85 nm, respectively. Z-stacks with a step size of 150 nm were acquired and optimized using Nyquist Calculator (Scientific Volume Imaging). Images were deconvolved using Huygens Professional software (Scientific Volume Imaging). For STED images, single focal planes are shown, whereas the movie (Movie 1 available in Online Resource 2) shows a Z-stack. Data were analyzed using LAS Advanced Fluorescence 2.6.3 and ImageJ software. Line segments were drawn in a single focal plane to determine the intensity values plotted in the form of line graphs which show fluorescence intensity in AU. GVBs for which the quality of the image was sufficient to classify them as single, multiple or filling core GVB were included for the quantification of GVB morphology types.

### Immunocytochemistry followed by transmission electron microscopy (TEM)

For TEM, neurons were blocked and permeabilized with 2% goat serum (Gibco) and 0.05% saponin (Sigma) in PBS (blocking buffer) for 30 min at RT. Neurons were incubated with the primary antibody CK1δ diluted in blocking buffer (1:100) o/n incubation at 4 °C (Table 1), followed by PBS washes and incubation with biotinylated goat-anti-mouse secondary antibody diluted in blocking buffer (1:400) (Jackson ImmunoResearch) for 90 min at RT. After washing with PBS, neurons were incubated for 90 min at RT to form the avidin–biotin HRP complex using a Vectastain ABC kit (Vector Laboratories) and subsequently washed with PBS and developed using the chromogen DAB (DAB Substrate Kit, Vector Laboratories) according to manufacturer’s instructions.

Neurons were post-fixed in 1% osmium tetroxide and 1.5% potassium ferricyanide and subsequently dehydrated through increasing ethanol concentrations (30%, 50%, 70%, 90%, 96%, 100%), followed by embedding in epoxy resin. Resin blocks were cut to 80 nm ultrathin sections which were captured on formvar-coated copper grids. Sections were contrasted by 0.5% uranyl acetate and Reynolds lead citrate and analyzed by TEM in a JEOL1010 electron microscope (JEOL, Japan). Images were acquired at 30,000 × magnification using a side-mounted CCD camera (Morada; Olympus Sof Imaging Solutions) and iTEM analysis software (Olympus Sof Imaging Solutions). ImageJ software was used to adapt images for publication.

### Immunoblot analysis

Cells were washed in ice-cold PBS and scraped into ice-cold lysis buffer consisting of 1% Triton in PBS supplemented with phosphatase inhibitor (Sigma-Aldrich) and protease inhibitor (Roche) cocktail. Lysates were cleared by centrifugation for 20 min at 22,000*g* at 4 °C. Protein concentration in the supernatant was determined using a bicinchoninic acid (BCA) protein assay (Pierce). Protein concentration and loading volume were equalized among samples.

Sequential extractions were performed to separate lysates into a Triton X-100 soluble and insoluble fraction. After scraping cells in ice-cold 1% Triton lysis buffer supplemented with phosphatase inhibitor and protease inhibitor, lysates were spun down at 100,000*g* for 30 min at RT in a Sorvall M120-SE ultracentrifuge with Sorvall S100-AT6 rotor. The supernatant was kept as the Triton X-100 soluble fraction. The pellet was washed in lysis buffer and spun down using the same ultracentrifugation step. The resulting pellet was resuspended in 1% sodium dodecyl sulfate (SDS) in PBS supplemented with phosphatase inhibitor and protease inhibitor cocktail and the supernatant obtained by ultracentrifugation. The supernatant was kept as the Triton X-100 insoluble fraction. BCA protein assay was used to determine protein concentration in the Triton X-100 soluble fraction. Of the corresponding insoluble fraction threefold more volume was loaded.

Loading buffer containing SDS was added to the lysates and samples were boiled for 5 min at 96 °C. Samples were separated on 10% or 4–15% gradient (for sequentially extracted samples) Mini-Protean TGX stain-free precast polyacrylamide gels (Bio-Rad) and transferred onto nitrocellulose membranes (Bio-Rad). Membranes were blocked in 5% milk (Merck) in TBS containing 0.05% Tween (TBS-T) (Merck) and subsequently probed with primary antibodies diluted in blocking buffer o/n at 4 °C, with exception of GAPDH antibody which was incubated for 2 h at RT. Primary antibodies and dilutions are listed in Table 1. Antibody incubations were performed sequentially. After TBS-T washes, membranes were incubated with HRP-conjugated secondary antibodies (DAKO) diluted in blocking buffer (1:2000) for 1 h at RT, washed in TBS-T and developed using Lumi-Light Western blotting substrate (Roche) or SuperSignal West Femto substrate (Thermo Scientific). Chemiluminescence was visualized with the Odyssey Fc system (Li-Cor) and analyzed using Image Studio 5.2 software (Li-Cor). The total amount of protein loaded was visualized on the membrane using the Gel-Doc EZ system (Bio-Rad).

### Statistics

GraphPad Prism 6.0 software was used for statistical analysis. Shapiro–Wilk test was used to assess distribution normality. If the assumptions of normality were met, the parametric Student’s *t* test or one-way ANOVA followed by Tukey’s multiple comparison post hoc test was used. Otherwise, the non-parametric Mann–Whitney *U* or Kruskal–Wallis followed by Dunn’s multiple comparison post hoc test was used. One-sample *t* test was used to test the percentage of neurons with tau pathology and GVBs in PFF-injected mice against the value 0 (representing the 0% positive neurons observed in the control animals). Correlation was analyzed by parametric Pearson correlation analysis. A *p* value of ≤ 0.05 was considered statistically significant.

## Results

### Seeding of tau pathology induces GVBs in vivo

**Fig. 1:**
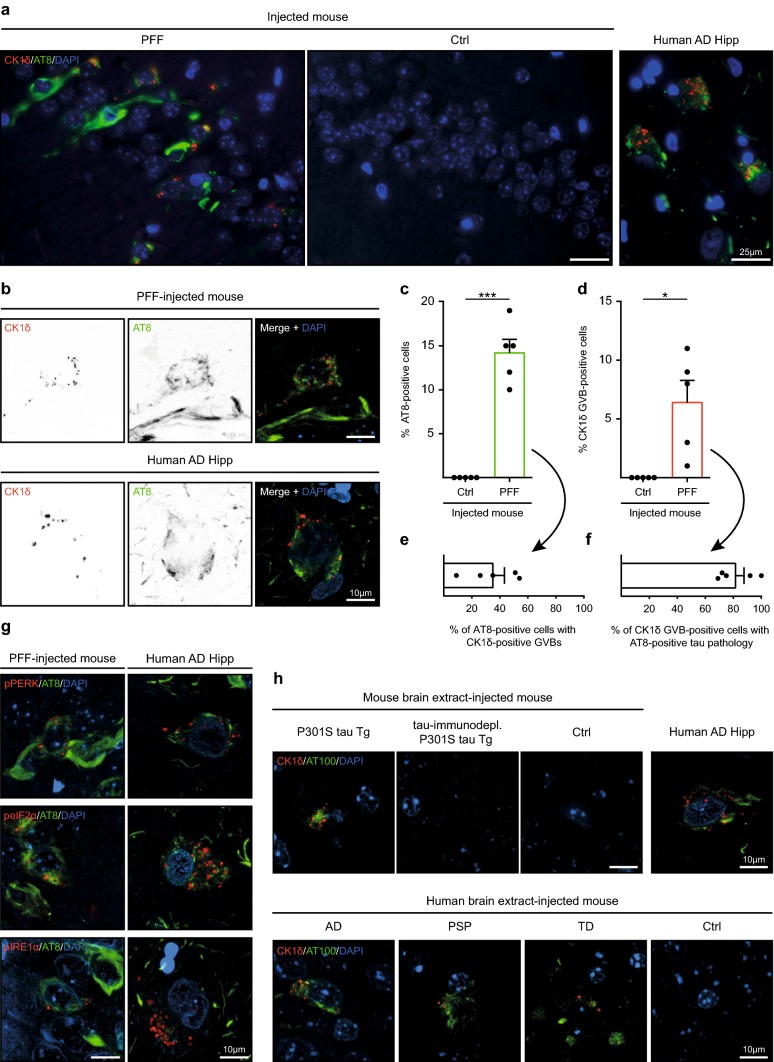
Seeding of tau pathology induces GVBs in vivo. **a**–**g** Immunofluorescence was performed on hippocampal sections of tau P301L Tg mice injected with K18 tau P301L seeds (PFF, *N *= 5) or control buffer (Ctrl, *N *= 5). **a** Representative epifluorescence images of immunostaining using AT8 (green) and the GVB marker CK1δ (red). Note that the AT8 epitope is absent from K18 PFFs. **b** Representative confocal images of neurons positive for immunolabeling with AT8 and CK1δ (shown separately in grayscale and in color in the merge: AT8 in green and CK1δ in red). **c**–**f** Quantification of **a** showing the percentage of pyramidal neurons positive for AT8 (**c**) or CK1δ (**d**) in the hippocampal CA1, CA2 and CA3 area of Ctrl or PFF-injected mice. *p < 0.05, ***p < 0.001, one-sample *t* test. **e** Quantification of the percentage of AT8-immunoreactive neurons that contains CK1δ-labeled GVBs and **f** of neurons with CK1δ-positive GVBs that is also positive for AT8 in the hippocampus of PFF-injected mice. Bars indicate the mean + SEM. Data points represent (mean values of) individual animals. See Supplementary Table 2 in Online Resource 1 for an overview of the number of cells analyzed. **g** Representative confocal images of neurons positive for immunostaining with AT8 (green) and the GVB markers pPERK, peIF2α and pIRE1α (red). **h** ALZ17 mice injected with extract from mouse brain [P301S tau Tg (*N *= 6), tau P301S Tg immunodepleted of tau (tau-immunodepl. P301S tau, *N *= 4), non-Tg C57BL/6 mice (Ctrl, *N *= 1)] or human post-mortem brain [AD (*N *= 6), PSP (*N *= 3), TD (*N *= 4) patients or control (Ctrl, *N *= 4)]. Representative confocal images of immunofluorescence with AT100 (green) and CK1δ (red) in the injected mice are shown. Human AD hippocampus (Human AD Hipp) is included for reference in all immunostainings. Cell nuclei are stained with DAPI (blue) in all images

We investigated the presence of GVBs in animal models of seeded tau aggregation. We used an established murine tau seeding model that employs injection of recombinant K18 tau P301L PFFs or control buffer into the hippocampus of tau P301L Tg mice at 3 months of age [52]. As previously reported, injection with PFFs, but not control buffer, resulted in tau pathology positive for the phospho-specific tau antibody AT8 at 2 months post-injection (Fig. 1a–c). In addition, in neurons in the hippocampi of PFF-injected mice, puncta positive for the GVB marker CK1δ were detected, whereas these were absent from control buffer-injected mice (Fig. 1a, b, d). These punctate structures in the PFF-recipient mice were also immunoreactive for other human GVB markers: pPERK, peIF2α and pIRE1α (Fig. 1g). Hence, seeding of tau pathology in vivo using synthetic tau seeds induces GVBs, immunoreactive for established GVB markers.

To elaborate on the connection between tau pathology and the presence of GVBs, double immunofluorescence was used to quantify the number of neurons immunoreactive for either AT8, CK1δ or both in the hippocampi of the PFF-treated mice. The vast majority (80%) of neurons with CK1δ-labeled GVBs contained AT8-positive tau aggregates (Fig. 1f). In contrast, only 35% of neurons with AT8-positive tau aggregates presented GVBs (Fig. 1e). Quantification using pPERK instead of CK1δ as GVB marker yielded similar results (Supplementary Fig. 1 in Online Resource 1). Therefore, most neurons that have GVBs also have tau pathology, but not vice versa.

We extended these findings to an observational study of the presence of GVBs in material obtained from two previously published studies that employ mouse or human brain extracts to seed tau pathology in mice [12, 13]. In the first model, brain lysates of tau P301S Tg mice with filamentous tau pathology or of age-matched non-Tg C57BL/6 mice were injected into the hippocampus and overlaying cerebral cortex of WT tau ALZ17 mice [13]. In the second model, tau pathology was seeded in ALZ17 mice by injection with human post-mortem brain lysate from donors with confirmed neuropathology [12] (Supplementary Fig. 2a, Supplementary Table 1 in Online Resource 1). As previously reported, injection with tau P301S Tg mouse brain lysate induced tau pathology positive for the phospho-tau specific AT100 antibody (Fig. 1h). Tau pathology was absent from animals injected with non-Tg control mouse brain lysate. Likewise, brain extracts of human AD and primary tauopathy (PSP and TD) patients, but not from an age-matched control, induced AT100-positive tau aggregates (Fig. 1h). In all these seeded tau aggregation models, GVBs immunoreactive for CK1δ (Fig. 1h) and pPERK (Supplementary Fig. 2b in Online Resource 1) were detected in neurons in mice where injection with brain homogenate resulted in tau pathology. GVBs were observed using tau P301S Tg mouse as well as human tauopathy patient brain extract as source of tau seeds, whereas GVBs were absent in mice injected with control mouse or human brain lysate (Fig. 1h; Supplementary Fig. 2b in Online Resource 1). To rule out that other factors than tau present in brain lysate induce GVBs, ALZ17 mice were injected with tau P301S Tg mouse brain lysates immunodepleted of tau [13]. In these mice, neither AT100-positive tau pathology nor GVBs were detected (Fig. 1h; Supplementary Fig. 2b–d in Online Resource 1). Together, these results show that seeding tau pathology with PFFs of recombinant mutant tau fragments as well as with full length WT and mutant tau seeds present in brain tissue leads to the formation of GVBs in vivo.

### Seeding of tau pathology induces GVBs in cultured primary neurons

To investigate whether the seeding of tau pathology also induces GVBs in vitro, we established the equivalent of the in vivo recombinant tau seeding model in primary mouse neurons. To this end, recombinant K18 tau P301L PFFs or control buffer were added for 11 days to the culture medium of hippocampal neurons with lentivirus-mediated expression of fluorescently tagged human tau P301L (Supplementary Fig. 3a, b in Online Resource 1). Treatment with PFFs, but not control buffer, resulted in re-localization of tau into aggregates that were recognized by AT100 as well as by the MC1 antibody that binds a pathological conformation of tau (Supplementary Fig. 3c, d in Online Resource 1). The seeding-induced tau aggregates were detergent-insoluble and resistant to MeOH extraction of soluble proteins (Supplementary Fig. 3e, f in Online Resource 1). Neither lentiviral tau transduction nor PFF exposure increased cell death (Supplementary Fig. 3g in Online Resource 1). In conclusion, as previously reported [27], tau seeding in cultured primary neurons expressing human mutant tau induces tau pathology.

**Fig. 2:**
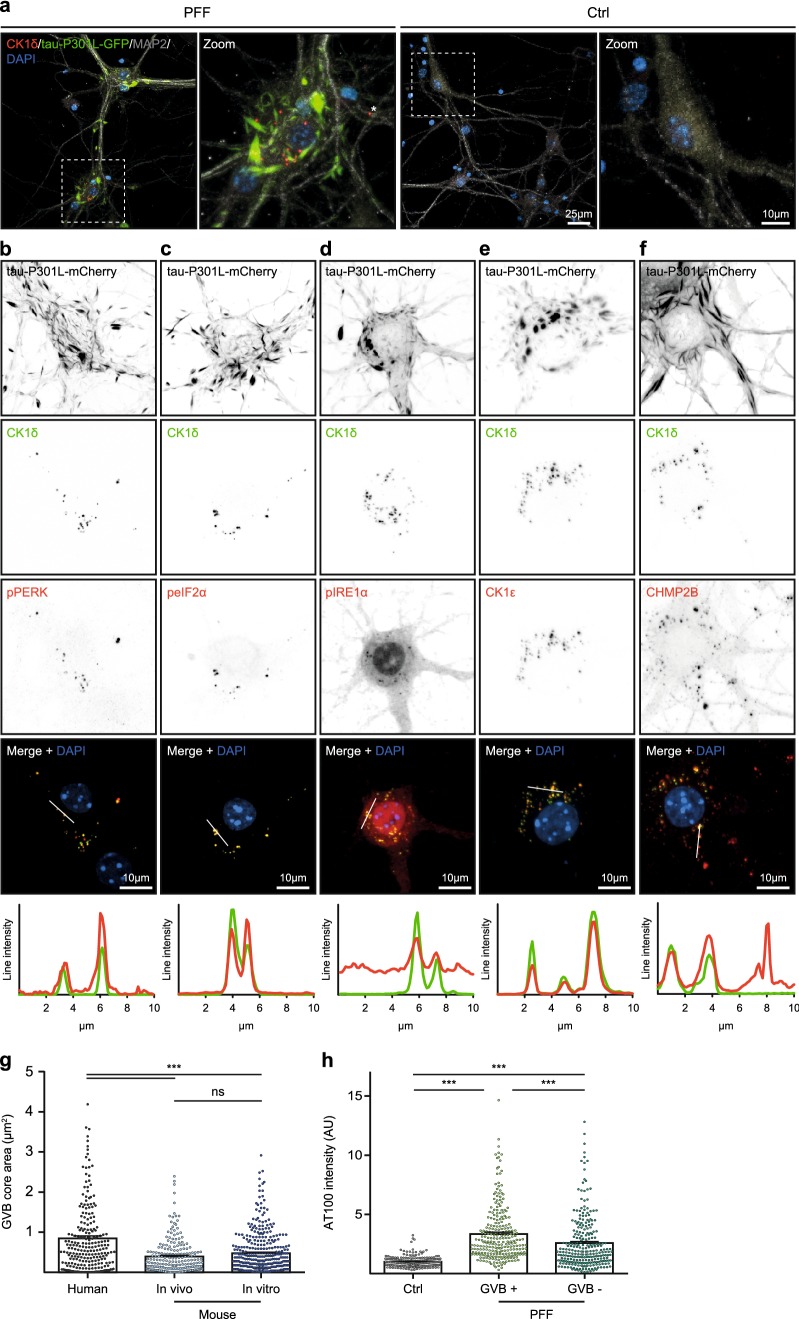
Seeding of tau pathology induces GVBs in cultured primary neurons. **a** Representative confocal images and zooms of primary mouse hippocampal neurons expressing tau-P301L-GFP (green) treated with K18 tau P301L seeds (PFF) or control buffer (Ctrl). Neurons were immunostained with the GVB marker CK1δ (red) and the neuron-specific dendritic marker MAP2 (gray). Zoomed areas are indicated in the merged image. Asterisk marks a dendritic GVB. Images were acquired with optimal settings for aggregated tau. The expression of the tau transgene acquired with optimal settings for non-aggregated tau is visualized in Supplementary Fig. 3a in Online Resource 1. **b**–**f** Representative confocal images and fluorescence intensity profiles of PFF-treated tau-P301L-mCherry expressing primary mouse neurons showing the overlap of CK1δ with the additional GVB markers pPERK (**b**), peIF2α (**c**), pIRE1α (**d**), CK1ɛ (**e**) or CHMP2B (**f**). Individual fluorescence signals are shown separately in grayscale and in color in the merge: CK1δ in green and the other GVB markers in red. Tau-P301L-mCherry signal is not shown in the merge. Intensity profiles are determined along the line segment shown as a white bar in the merge. Cell nuclei are stained with DAPI (blue) in all images. See Supplementary Table 3 in Online Resource 1 for an overview of the number of cells analyzed. **g** Quantification of the area of the CK1δ-labeled GVB core in hippocampal neurons in the human AD brain (human) and in the PFF seeding models in vivo (mouse—in vivo) and in vitro (mouse—in vitro). Note that a single high value in the human AD brain group was taken along in the analysis but is not shown in the graph. Data points represent individual GVBs. *N* ≥ 3 independent experiments, *N* = 237, 215 and 367 GVBs analyzed for human, mouse—in vivo and mouse—in vitro, respectively. ***p < 0.001, *ns* not significant, Kruskal–Wallis test followed by Dunn’s multiple comparison test. **h** Quantification of the fluorescence intensity of immunolabeling with AT100. Ctrl cells were randomly selected, in PFF-treated cultures (PFF) two populations were analyzed: cells with GVBs (GVB +) and cells without GVBs (GVB −). Data points represent individual neurons. *N* = 3 independent experiments, *N* = 230, 257 and 260 neurons analyzed for Ctrl, GVB + and GVB −, respectively. ***p < 0.001, Kruskal–Wallis test followed by Dunn’s multiple comparison test. Bars indicate the mean + SEM

Immunostaining using the GVB marker CK1δ revealed intensely labeled punctate structures in a subset of the PFF-treated neurons that primarily localized to the neuronal soma, although occasionally they were also detected in proximal dendrites (Fig. 2a). Double immunofluorescence was performed for additional GVB core markers: pPERK, peIF2α, pIRE1α, CK1ɛ and CHMP2B. Immunoreactivity for all these markers overlapped with the CK1δ-positive punctate structures (Fig. 2b–f). Using this panel of GVB markers, GVBs were not detected in control buffer-treated cultures (Fig. 2a, data not shown). Hence, the seeding of tau pathology induces GVBs in vitro that are positive for common GVB markers.

To better characterize GVBs, the size of the CK1δ-positive GVB core in hippocampal neurons was determined in the in vitro and in vivo recombinant tau seeding models and in the human AD brain. The area of the CK1δ-labeled GVB core averaged 0.45 µm^2^ in primary hippocampal neurons, which did not differ significantly from the 0.40 µm^2^ measured in neurons in mouse hippocampus (Fig. 2g). The mean area of CK1δ-immunoreactive GVB cores in hippocampal neurons in the AD brain was 0.80 µm^2^ and significantly larger than in mouse neurons in vitro and in vivo (1.8- and 2.0-fold, respectively). However, as the soma size of GVB-containing neurons in human hippocampus was also significantly larger (2.3-fold) than in mouse hippocampus (Supplementary Fig. 4 in Online Resource 1), the area occupied by GVB cores is proportional to the size of the neuronal soma.

To test if neurons with GVBs in vitro also have tau pathology, the AT100 fluorescence intensity was analyzed by confocal microscopy in control neurons and in GVB-positive and -negative neurons in PFF-treated cultures. PFF-treated neurons with CK1δ-positive GVBs had a significantly higher AT100 signal intensity compared to randomly sampled neurons in the control condition (Fig. 2h). The AT100 intensity in PFF-treated neurons without GVBs was also significantly increased compared to control cells (Fig. 2h). These data show that GVBs form in a subset of neurons with tau pathology.

### Intracellular tau pathology causes GVB formation

**Fig. 3:**
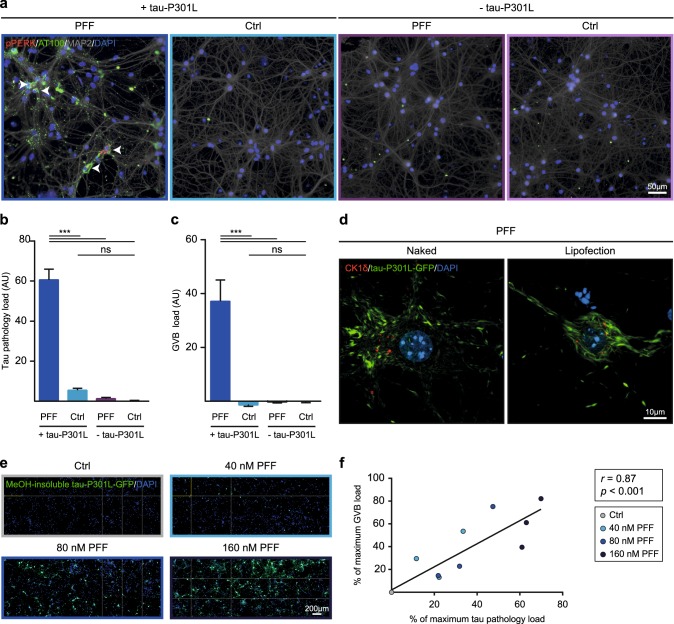
Intracellular tau pathology causes GVB formation. **a**–**c** Primary mouse neurons with (+) or without (−) expression of tau-P301L in the presence (PFF) or absence (Ctrl) of seeds. Representative epifluorescence images of immunostaining with AT100 (green), the GVB marker pPERK (red) and the neuron-specific dendritic marker MAP2 (gray) are shown. Image acquisition and analysis were automated. Arrowheads point to examples of neurons with GVBs. **b**, **c** Quantification of the conditions shown in **a**. Tau pathology load (**b**) and GVB load (**c**) were quantified as described in the “Materials and methods” and are presented as normalized background corrected values. Bars indicate the mean of the observations + SEM. *N* = 3 independent experiments, *N* = 16 observations per condition, on average 6101 neurons per observation. ***p < 0.001, *ns* not significant, tau pathology load: one-way ANOVA followed by Tukey’s multiple comparison test, GVB load: Kruskal–Wallis test followed by Dunn’s multiple comparison test. **d** Representative confocal images of tau-P301L-GFP (green) expressing neurons immunostained with the GVB marker CK1δ (red) that were exposed to PFFs either by direct addition to the culture medium (Naked) or by Lipofectamine 2000-mediated transduction (Lipofection). See Supplementary Table 3 in Online Resource 1 for an overview of the number of cells analyzed. **e**, **f** Neurons expressing tau-P301L-GFP (green) were treated with 40, 80 or 160 nM PFFs or control buffer (Ctrl). At the endpoint of the experiment soluble tau was extracted using ice-cold MeOH. Image acquisition and analysis were automated. **e** Representative example showing 24 representative epifluorescence images per condition. **f** Quantification of the tau pathology and GVB load in the conditions shown in **e** and measured as described in the “Materials and methods”. Data points represent average of *N* = 16–18 observations from *N* = 3 independent experiments, on average 7747 neurons per observation. ***p < 0.001, Pearson *r* = 0.87. Linear regression line is shown in the graph and inset shows *r* and *p* value. Cell nuclei are stained with DAPI (blue) in all images. Quantification of cell number in the conditions shown in **a** and **e** are shown in Supplementary Fig. 3g and Supplementary Fig. 5b in Online Resource 1, respectively

Automated microscopy was employed to further investigate the causality between tau pathology and GVBs. To determine which step in the seeded tau aggregation process results in GVB formation, PFFs were added to neurons with or without human tau P301L expression. As described above, PFF treatment induced AT100-positive tau pathology and GVBs in neurons that express tau P301L (Figs. 2, 3a–c; Supplementary Fig. 3c in Online Resource 1). In contrast, PFF addition to neurons without tau P301L expression induced neither tau pathology nor GVBs (Fig. 3a–c). Therefore, the development of tau pathology rather than seed exposure induces GVB formation.

In the experiments described thus far PFFs were delivered as naked protein, probably entering the cell via an endocytic pathway. This physiological internalization route was circumvented by Lipofectamine-mediated transduction, which facilitates delivery of the PFFs to the cytosol via lipofection. PFF transduction by lipofection induced both tau pathology and GVBs in neurons (Fig. 3d). Hence, irrespective of the seed uptake route, GVBs develop when intracellular tau pathology is present.

Finally, the addition of increasing concentrations of PFFs resulted in a dose-dependent increase of MeOH-insoluble intracellular tau pathology, in the absence of cell death (Fig. 3e, f; Supplementary Fig. 5 in Online Resource 1). Quantification of the number of neurons with GVBs in this experiment demonstrated a strong positive correlation (*r *= 0.87) between the tau pathology and GVB load (Fig. 3f; Supplementary Fig. 5a in Online Resource 1). This result strengthens the conclusion that GVBs develop as a consequence of intracellular tau pathology.

Under physiological conditions, tau binds to microtubules to regulate their stability and dynamics and intracellular tau aggregation is accompanied by disturbed microtubule structure and function (for review see Ref. [8]). To test whether microtubule disruption underlies GVB development, neurons were treated with the microtubule-destabilizing agent vinblastine. Vinblastine treatment resulted in a distorted staining pattern of β-III-tubulin and the stable microtubule marker acetylated α-tubulin, indicative of microtubule disruption (Supplementary Fig. 6 in Online Resource 1). No punctate structures positive for the GVB marker CK1δ were detected upon vinblastine treatment (Supplementary Fig. 6 in Online Resource 1). Hence, microtubule disruption per se does not induce GVB formation.

### GVB formation is a neuron-selective process

**Fig. 4:**
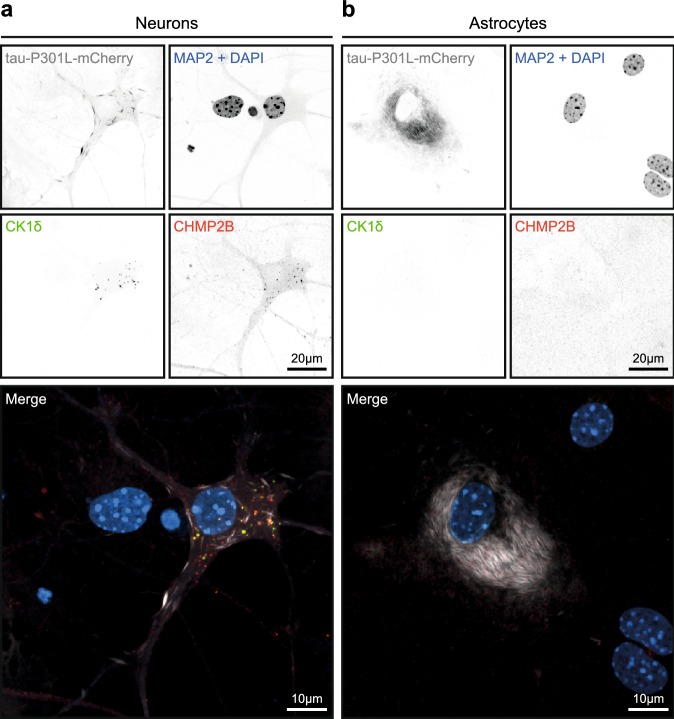
GVBs are not detected in cultured primary astrocytes with seeding-induced tau pathology. **a**, **b** Primary mouse neurons (**a**) and astrocytes (**b**) expressing tau-P301L-mCherry were cultured separately and tau pathology was seeded using PFFs. Representative confocal images of neurons and astrocytes immunolabeled with the GVB markers CK1δ and CHMP2B and the neuron-specific dendritic marker MAP2 are shown. Individual fluorescence signals are shown separately in grayscale and in color in the merge: CK1δ in green, CHMP2B in red, tau-P301L-mCherry in gray and MAP2 and the nuclear stain DAPI both in blue. Whole coverslips with near confluent astrocytes of *N* = 3 independent experiments were analyzed for the presence of GVBs

Like tau pathology, GVBs are mainly observed in neurons in the human brain, although occasionally also in glial cells [50, 69]. To investigate whether a similar selectivity exists in vitro, the same tau seeding paradigm was applied to non-neuronal cells. Primary mouse astrocytes expressing fluorescently tagged human tau P301L were exposed to PFFs. As in neurons, PFF treatment in astrocytes resulted in the formation of detergent- and MeOH-insoluble intracellular tau aggregates labeled by AT100 and MC1 (Supplementary Fig. 7 in Online Resource 1). In contrast to neurons, punctate structures double positive for the GVB markers CK1δ and CHMP2B were not detected upon PFF-induced tau seeding in astrocytes (Fig. 4). Similar results were obtained upon the seeding of tau pathology with PFFs in HEK293 cells (Supplementary Fig. 8 in Online Resource 1). Western blotting confirmed the expression of CK1δ and CHMP2B in both cell types (Supplementary Fig. 9 in Online Resource 1). These data indicate that GVB formation in vitro is a neuron-selective process.

In brains of PSP patients, tau pathology is present in neurons as well as in astrocytes. Interestingly, in ALZ17 mice injected with PSP brain extract, tau aggregation is not only observed in neurons but also occasionally in astrocytes in structures resembling tufted astrocytes [12]. GVBs were abundantly present in neurons with tau pathology in these mice (Fig. 1h; Supplementary Fig. 2b, e in Online Resource 1), whereas only a single astrocytic GVB was observed (Supplementary Fig. 2e in Online Resource 1). This suggests that also in vivo tau-induced GVB formation predominantly occurs in neurons.

In the human AD brain, GVBs form predominantly in the hippocampus and in later GVD stages in the cortex and other brain areas, following the spreading pattern of tau pathology [67]. To study whether this regional preference of GVBs is recapitulated in vitro, tau pathology was seeded using PFFs in tau P301L expressing primary neuronal cultures derived from mouse hippocampus, cortex and striatum. Upon the seeding of tau pathology in cortical cultures, GVBs positive for the GVB markers CK1δ and pPERK were detected in neurons with tau pathology (Supplementary Fig. 10a). Analysis by automated microscopy showed that GVBs were present to a similar extent in cortical and hippocampal neurons with seeded tau aggregation (Supplementary Fig. 10b). Furthermore, CK1δ- and pPERK-positive GVBs formed in neurons with tau pathology derived from the striatum (Supplementary Fig. 10a), a region that is relatively spared from GVD in the AD brain [67]. Together, these data suggest that neurons in vitro can form GVBs in response to tau pathology irrespective of the brain region from which they originate.

### GVBs are positive for late autophagic and endocytic markers

**Fig. 5:**
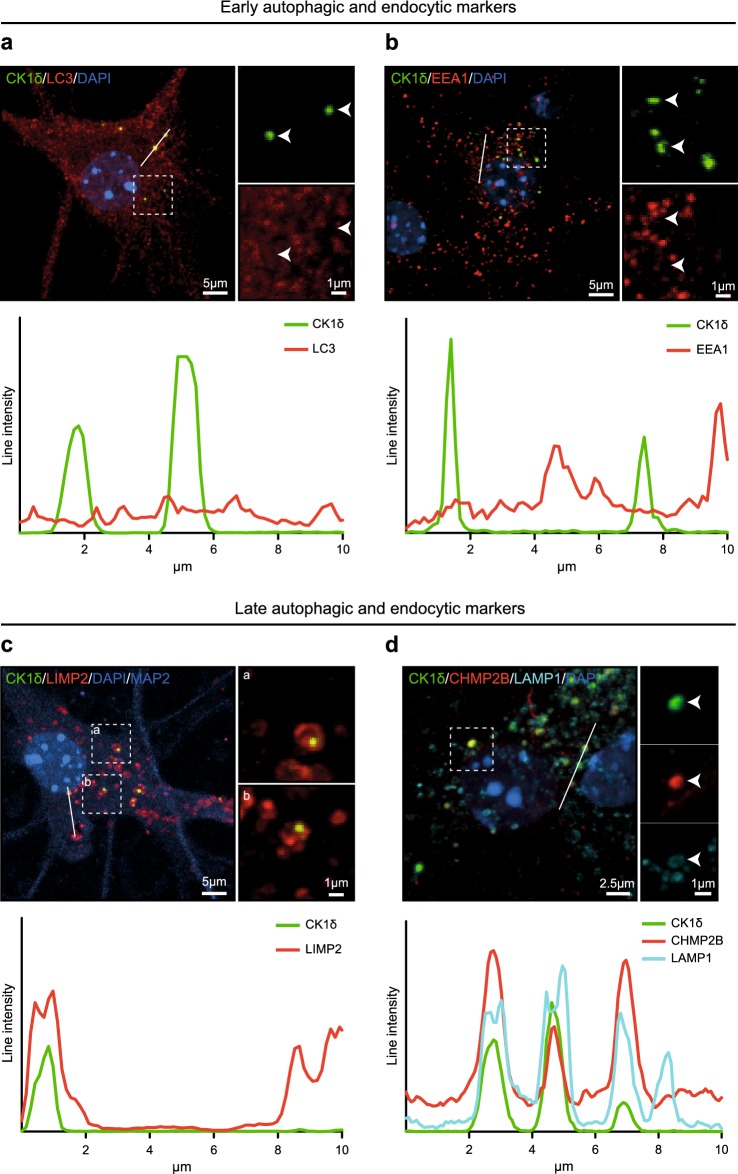
Tau-induced GVBs are positive for late autophagic and endocytic markers. Tau pathology was induced in primary mouse neurons expressing tau P301L by treatment with PFFs. **a**–**c** Representative confocal images plus zooms and fluorescence intensity profiles of immunofluorescent stainings using the GVB marker CK1δ (green) and the autophagosome marker LC3 (**a**), the early endosome marker EEA1 (**b**) or the late autophagic and endocytic membrane marker LIMP2 (**c**) (red). **d** Shows CK1δ (green) with the late autophagic and endocytic membrane marker LAMP1 (cyan) plus late endosomal marker CHMP2B (red). Zoomed areas are indicated in the merged image. Intensity profiles are determined along the line segment shown as a white bar in the merge. Cell nuclei are stained with DAPI (blue) in all images. In **c** the neuron-specific dendrite marker MAP2 is also shown in blue. MAP2 is not shown in the zooms. See Supplementary Table 3 in Online Resource 1 for an overview of the number of cells analyzed

To investigate the identity of the tau-induced GVBs, the overlap between the GVB marker CK1δ and proteins labeling different intermediates of the autophagic and endocytic pathways was studied by double immunofluorescence (see Fig. 9 for a schematic representation of these pathways and markers). To detect early autophagic and endocytic compartments, the prototypical markers LC3 and EEA1 were used, respectively. The LC3 antibody detects both cytosolic LC3-I and its lipidated form LC3-II that is recruited to autophagosomes [34, 46] and accordingly stains bafilomycin A1-induced LC3-positive punctate structures in primary mouse neurons (Supplementary Fig. 11g in Online Resource 1). In tau-seeded primary hippocampal neurons, LC3 immunolabeling showed a diffuse staining pattern. CK1δ-labeled GVBs were not LC3-positive (Fig. 5a). Immunolabeling for the early endosome marker EEA1 yielded a punctate staining pattern as expected. These EEA1-labeled structures did not co-localize with CK1δ-positive GVBs (Fig. 5b). Together, these data show that GVBs do not contain marker proteins of early autophagic and endocytic organelles.

Late autophagic and endocytic organelles comprise late endosomes (also known as multivesicular bodies) and amphisomes (resulting from fusion of autophagosomes with late endosomes) that ultimately both can fuse with lysosomes [7, 21, 36]. To detect these late stage organelles, immunolabeling for the transmembrane proteins LIMP2 and LAMP1 was performed. LIMP2 and LAMP1 showed overlapping immunoreactivity in ring-like structures and smaller puncta in neurons (Supplementary Fig. 12a in Online Resource 1). Double immunolabeling showed an association of LIMP2 and LAMP1 staining with GVBs: ring-like LIMP2 and LAMP1 immunoreactivity surrounded the CK1δ-positive core of GVBs (Fig. 5c, d). Furthermore, the late endosomal protein and established GVB marker CHMP2B co-localized with CK1δ inside the LAMP1-enclosed core of GVBs (Fig. 5d). Together, these data demonstrate that the GVB core and membrane contain late autophagic and endocytic markers.

No overlap was found between CK1δ-positive GVBs and the Golgi marker Golgi reassembly-stacking protein 55 (GRASP55), the endoplasmic reticulum (ER) membrane marker calnexin and the glutamatergic and GABAergic synapse markers vesicular glutamate transporter 1 (VGLUT1) and vesicular GABA transporter (VGAT), respectively (Supplementary Fig. 11a–d in Online Resource 1). Furthermore, although the stress granule marker Rab-GTPase-activating protein SH3-domain-binding protein (G3BP) stained thapsigargin-induced stress granules in primary mouse neurons, CK1δ-labeled GVBs were not positive for G3BP (Supplementary Fig. 11e, f in Online Resource 1). These data confirm the specific labeling of GVBs by late autophagic and endocytic markers.

### GVBs are lysosomal structures that contain degraded endocytic cargo

**Fig. 6:**
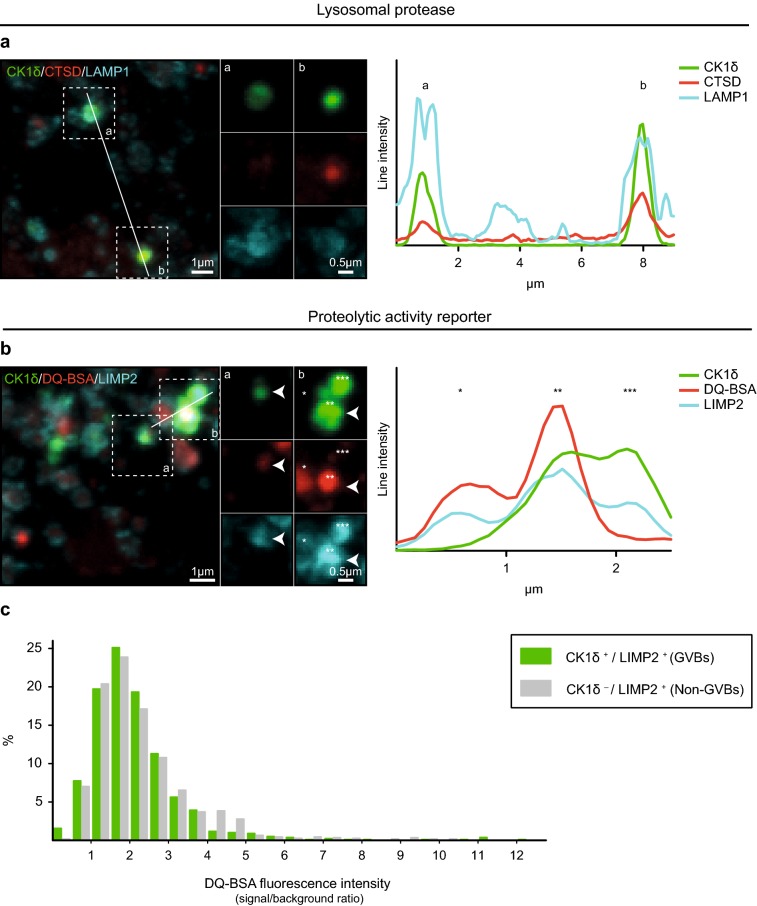
GVBs are lysosomal structures that contain degraded endocytic cargo. Tau pathology was induced in primary mouse neurons expressing tau P301L by treatment with PFFs. **a** Representative confocal images plus zooms and fluorescence intensity profiles of immunostaining with the GVB core marker CK1δ (green), the lysosomal protease CTSD (red) and the GVB membrane marker LAMP1 (cyan). See Supplementary Table 3 in Online Resource 1 for an overview of the number of cells analyzed. **b** Representative confocal images plus zooms and fluorescence intensity profiles of immunostaining with CK1δ (green) and LIMP2 (cyan) and the live cell reporter DQ-BSA (red). DQ-BSA fluorescence designates sites of proteolysis. Zoomed areas are indicated in the merged image. Intensity profiles are determined along the line segment shown as a white bar in the merge. **c** Quantification of **b** showing the DQ-BSA fluorescence signal in LIMP2-delineated structures negative or positive for the GVB marker CK1δ (see “Materials and methods” for analysis details). Frequency distribution of the DQ-BSA fluorescence intensity signal/cytosolic background ratio is presented as the percentage of structures within the specified bin. Bin width is 0.5. *N* = 3 independent experiments, *N* = 760 CK1δ-positive (GVBs) and 1038 CK1δ-negative (non-GVBs) LIMP2-positive structures analyzed

Autophagic and endocytic pathways ultimately converge at lysosomes to degrade their respective cargos. Therefore, the presence of the lysosomal protease cathepsin D (CTSD) in tau-induced GVBs was analyzed. As expected, CTSD immunolabeling was observed within LAMP1-labeled membranes (Supplementary Fig. 12b in Online Resource 1). Moreover, CTSD immunoreactivity overlapped with CK1δ- and LAMP1-positive GVBs (Fig. 6a).

To visualize lysosomal proteolytic activity in situ, the proteolysis reporter DQ-BSA was supplied to the medium of live neurons. DQ-BSA reports proteolytic activity in organelles connected to the endocytic pathway by dequenching of its fluorophore upon hydrolysis (Supplementary Fig. 12c in Online Resource 1). DQ-BSA fluorescence was detected in LIMP2-decorated structures (Supplementary Fig. 12d in Online Resource 1). Furthermore, the DQ-BSA fluorescence signal overlapped with CK1δ- and LIMP2-labeled GVBs (Fig. 6b). Quantification of the DQ-BSA fluorescence intensity in GVBs showed that in 91% of GVBs DQ-BSA fluorescence above cytosolic background was detected. For 46% of GVBs this was more than twofold (Fig. 6c). The DQ-BSA intensities observed in GVBs were similar to those observed in non-GVB LIMP2-positive structures (93% above background and 49% twofold above background). This experiment employing a live cell tool demonstrates that the majority of GVBs contain degraded endocytic cargo.

### GVBs contain one or more dense cores delineated by a single membrane

**Fig. 7:**
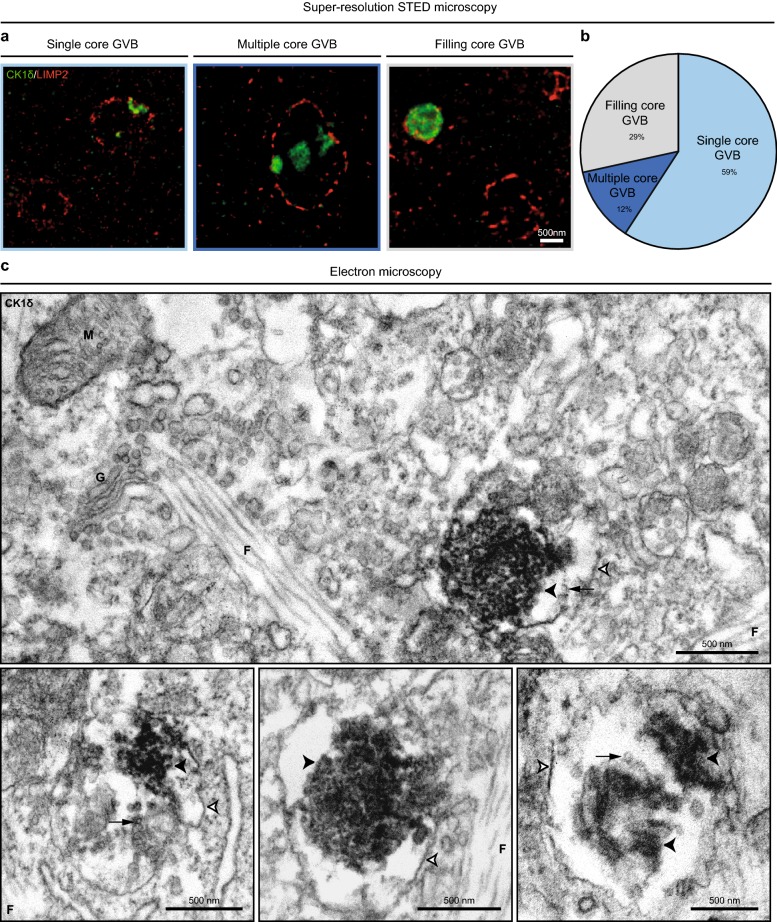
Tau-induced GVBs contain one or more cores enclosed by a single LIMP2-positive membrane. Tau pathology was induced in primary mouse neurons expressing tau P301L by treatment with PFFs and GVBs were visualized by super-resolution STED (**a**, **b**) or electron microscopy (**c**). **a** Representative super-resolution images obtained by STED microscopy showing different morphologies of GVBs immunolabeled for the GVB core marker CK1δ (green) and GVB membrane marker LIMP2 (red). The single core GVB is also displayed in Movie 1 available in Online Resource 2. The filled core GVB is also shown in Fig. 8c. **b** Quantification of the different GVB morphologies (single core, multiple core and filling core GVBs) detected in **a** (see “Materials and methods” for analysis details). *N* = 3 independent experiments, *N* = 137 GVBs quantified. **c** Electron micrographs of GVBs identified by CK1δ immunocytochemistry and DAB labeling. Visible are GVB cores (black arrowheads), a vacuole and a single limiting membrane (white arrowheads with black border). Small vesicular structures were frequently observed in the vacuole (black arrow). Mitochondrion (M), Golgi apparatus (G) and fibrillar structures (F) are indicated in the micrographs

The ultrastructure of tau-induced GVBs was investigated at high resolution by STED microscopy on neurons immunostained for the GVB core marker CK1δ and GVB membrane marker LIMP2. STED imaging showed that the majority of GVBs (59%) comprised a single CK1δ-positive GVB core and a clear vacuole enclosed by a LIMP2-decorated membrane (Fig. 7a, b; Movie 1 available in Online Resource 2). In 29% of GVBs the vacuolar space was absent as the CK1δ-positive core filled all space within the surrounding LIMP2-labeled membrane. A minority of GVBs (12%) contained multiple CK1δ-positive cores within a vacuole surrounded by a LIMP2-positive membrane (Fig. 7a, b). In conclusion, the morphology of GVBs is heterogeneous.

The ultrastructure of the tau-induced GVBs was further examined by electron microscopy. In order to identify the GVBs in the TEM images, CK1δ immunocytochemistry was performed using DAB labeling. Vesicular structures containing one or multiple granular core(s) positive for CK1δ were detected in a subset of PFF-treated neurons (Fig. 7c; Supplementary Fig. 13a in Online Resource 1), but not in control buffer-treated neurons (data not shown). The dense CK1δ-labeled core was surrounded by a clear vacuole. Small vesicular structures were frequently observed within the vacuole. The core and vacuole were surrounded by a single limiting membrane (see membrane zooms in Supplementary Fig. 13b in Online Resource 1). These CK1δ-positive GVBs were frequently observed in proximity to fibrillar structures resembling filamentous tau. No such fibrillar structures were detected within the GVB-delineating membrane. In conclusion, GVBs have one or multiple CK1δ-positive cores which are enclosed by a single limiting membrane.

### GVBs accumulate specific cytosolic cargo in the core

**Fig. 8:**
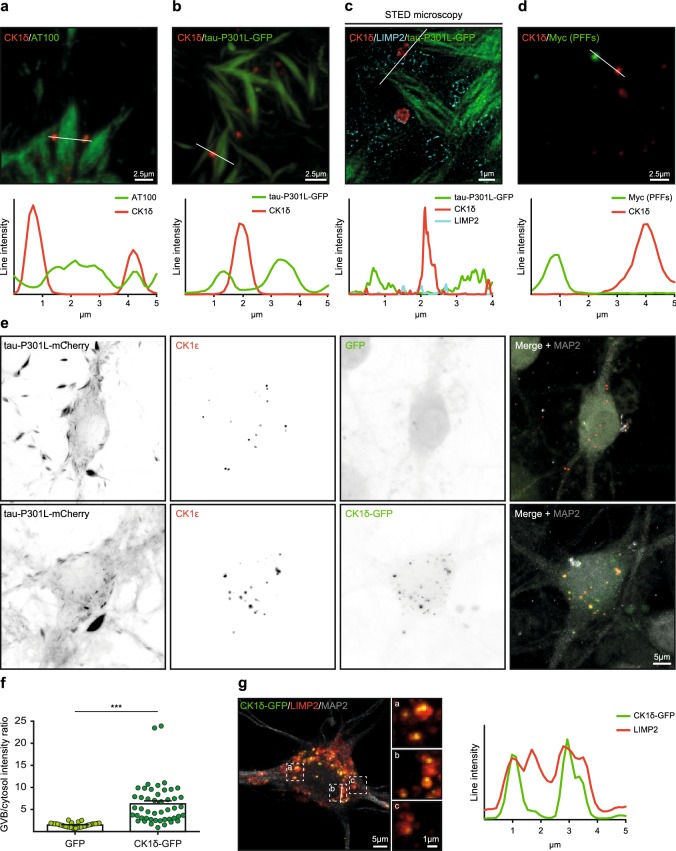
Specific cytosolic cargo accumulates in the core of GVBs. Tau pathology was induced in primary mouse neurons expressing tau P301L by treatment with PFFs. **a**, **b** Representative confocal images and fluorescence intensity profiles of immunostaining using the GVB core marker CK1δ (red) with AT100 staining (**a**) or with direct fluorescence signal from tau-P301L-GFP (**b**) (green). **c** Representative super-resolution image obtained by STED microscopy showing tau-P301L-GFP (green), CK1δ (red) and the GVB membrane marker LIMP2 (cyan). One of the GVBs in this image is also shown in Fig. 7a. **d** Representative confocal image and fluorescence intensity profile of immunostaining using CK1δ (red) plus Myc staining (green) to visualize the PFFs. **e** Representative confocal images showing tau-P301L-mCherry co-expressed with either GFP or CK1δ-GFP. Neurons were immunostained using the GVB marker CK1ɛ and the neuron-specific dendrite marker MAP2. Individual fluorescence signals are shown separately in grayscale and in color in the merge: GFP and CK1δ-GFP in green and CK1ɛ in red. MAP2 is shown in gray in the merge. Tau-P301L-mCherry signal is not shown in the merge. **f** Quantification of **e** showing the GVB/cytosol fluorescence intensity ratio of GFP and CK1δ-GFP fluorescence. *N *= 3 independent experiments, *N* = 34 and 47 neurons analyzed for GFP and CK1δ-GFP, respectively. ***p < 0.001, Mann–Whitney *U* test. **g** Representative confocal image and fluorescence intensity profile of the expression of CK1δ-GFP (green) plus immunostaining for LIMP2 (red) and MAP2 (gray). MAP2 is not shown in the zooms. See Supplementary Table 3 in Online Resource 1 for an overview of the number of cells analyzed

Next, the content of the GVB core was investigated in more detail. Studies on the presence of (phospho-) tau epitopes in the GVB core in the human post-mortem brain have generated contradicting results (for review see Ref. [37]). Therefore, the overlap of tau-induced GVBs with AT100 immunoreactivity, the expressed GFP-tagged tau P301L and the tau seeds was analyzed. Occasional co-localization of AT100 with the CK1δ-positive GVB core was observed (Fig. 8a). However, confocal as well as super-resolution STED microscopy imaging of the direct fluorescence of the GFP-tagged tau P301L showed no accumulation of aggregated or diffuse tau-P301L-GFP in GVBs (Fig. 8b, c). Also when the signal intensity of the diffuse tau-P301L-GFP was increased, no accumulation in GVBs was observed (Supplementary Fig. 14 in Online Resource 1). In addition, double immunofluorescent labeling using antibodies against CK1δ and the Myc-tag present in the tau PFFs showed no overlap (Fig. 8d). Our data indicate that neither cytosolic tau nor the internalized tau seeds accumulate in GVBs.

In contrast to tau, the cytosolic protein CK1δ consistently accumulates in GVBs in the human brain (Fig. 1), mouse brain (Fig. 1) and primary mouse neurons (Fig. 2), suggesting selectivity in the accumulation of CK1δ in GVBs. To test this hypothesis, lentivirus-mediated expression of GFP-tagged CK1δ was used. In control neurons, CK1δ-GFP showed a cytoplasmic distribution as expected (Supplementary Fig. 15a in Online Resource 1). In neurons containing tau-induced GVBs, CK1δ-GFP fluorescence accumulated in bright puncta that overlapped with CK1ɛ-positive GVBs (Fig. 8e). In contrast, GFP alone was distributed diffusely throughout the cytosol in neurons with tau-induced GVBs, and did not accumulate in the large majority of GVBs (Fig. 8e). The GVB/cytosol fluorescence intensity ratio was significantly higher for CK1δ-GFP than for GFP (6.3 and 1.5, respectively) (Fig. 8f). The CK1δ-GFP positive GVBs were also immunoreactive for pPERK (Supplementary Fig. 15b in Online Resource 1). In addition, it was confirmed that the CK1δ-GFP labeled GVB core was delineated by a LIMP2-positive membrane (Fig. 8g). Together, these data demonstrate that CK1δ-GFP specifically accumulates in the core of GVBs.

## Discussion

In the present study, we investigated the formation of GVBs, an abundant yet poorly understood structure in the tauopathy brain. Our first aim was to establish models for GVB formation. We show that seeding tau pathology causes the formation of GVBs in vivo in mouse models and in vitro in primary mouse neurons, but not in astrocytes or HEK293 cells. Secondly, we used these models to gain more insight into the identity of GVBs. Our data demonstrate that GVBs are lysosomal structures in which endocytic and specific cytosolic cargo accumulates.

### Seeding-induced intracellular tau pathology triggers GVB formation independent of tau isoform, structure or mutations

The present study shows that upon seeding of tau pathology GVBs are formed in neurons with tau pathology in vivo and in vitro. We demonstrate a strong correlation between the extent of intracellular tau pathology and the GVB load in vitro. Importantly, our data indicate that exposure to seeds or other brain-derived factors per se does not lead to GVB formation. Therefore, we conclude that intracellular tau pathology causes the formation of GVBs.

In the human brain, GVBs are occasionally observed in neurons in which tau pathology is not detected [30, 50]. Also in our model GVBs are observed in a small subset of neurons without apparent tau pathology. However, we demonstrate GVB formation only in experimental conditions where intracellular tau pathology is induced, suggesting that GVBs can form in response to early pathological tau species. These may not be detected because they differ from the later stage aggregates (e.g., oligomeric rather than fibrillary tau assemblies) or they may be low abundant and therefore below the detection limit.

Previous studies have described GVBs in tau Tg mouse models [33, 38, 41, 45]. In these models GVBs are rare even after more than 9-months overexpression of the mutant tau transgene. We extend these findings to murine models of seeded tau pathology. In the in vivo PFF seeding model, GVBs are readily observed 2 months after seeding. Upon seeding of tau aggregation with PFFs in cultured neurons, GVBs are formed within 11 days. Therefore, the current study provides convenient experimental methods to induce and study GVB formation.

We report the induction of GVBs upon seeding with recombinant tau P301L PFFs and with tau fibrils derived from brains of AD, PSP and TD patients and tau P301S Tg mice, both in recipient neurons expressing WT and P301L mutant tau. These findings are in accordance with data from the human brain that describe: (1) GVBs in sporadic tauopathy patients as well as in *MAPT* mutation carriers; (2) GVBs in tauopathies characterized by the predominance of 3R or 4R tau isoforms or both in the aggregates [3, 39, 50, 64, 76]. Therefore, GVB formation is independent of the tau isoform or presence of pathogenic mutations.

In the majority of our experiments K18 tau PFFs were used. Recent cryo-EM studies have shown that brain-derived tau filaments differ structurally from heparin-induced tau filaments assembled in vitro [80] and that K18 tau PFFs lack ten residues from the sequence that forms the filament core in AD and Pick’s disease patient brains [17, 19]. Therefore, the intracellular aggregates induced by K18 tau PFFs may structurally differ from those in the human brain. The current study also demonstrates GVB induction upon seeding with brain-derived tau fibrils, indicating that GVB formation is independent of the seed structure.

Although many neurodegenerative diseases are characterized by protein aggregation, data from the human brain suggest a strong association of tau pathology with GVBs. Also in cases with Parkinson’s disease and multiple system atrophy, both characterized by intracellular aggregation of α-synuclein, GVBs have been described, although one study detected comorbid tau pathology in GVB-bearing neurons [28, 29, 43, 67, 77]. GVBs are not detected in patients with tau-negative FTD subtypes displaying intracellular deposition of fused in sarcoma or TAR DNA-binding protein 43 protein [50]. Furthermore, extracellular protein aggregation seems unrelated to GVB formation as GVBs are absent from mouse models of Aβ pathology [38, 45] and not detected in brains of prion disease patients, unless comorbid tau pathology is present [69, 75]. In conclusion, the causal relation between tau pathology and GVBs demonstrated in the present study is in line with their strong association in the human brain.

### Experimentally induced GVBs mimic GVBs in the human brain

In the in vitro model described here, GVBs are positive for CK1δ and CK1ɛ, which are commonly used to label GVBs in human neuropathological studies and form the basis of the Thal staging system for GVB distribution in the human brain [23, 67]. In addition, GVBs induced in our in vitro model are immunoreactive for other common GVB markers, including CHMP2B [78], the UPR activation markers pPERK, peIF2α and pIRE1α [30] and the GVB membrane marker LAMP1 [20]. In accordance, GVBs induced by the seeding of tau pathology in vivo were immunoreactive for CK1δ, pPERK, peIF2α and pIRE1α. Similar to the human brain, GVBs in the model systems described here mainly localize to the neuronal soma. Furthermore, the characteristic ultrastructural properties of GVBs in the human brain (for review see Ref. [37]) were recapitulated in the in vitro model. STED and electron microscopy showed the presence of a limiting membrane surrounding the vacuole and dense GVB core(s). The experimentally induced GVBs in mouse neurons are smaller than in the human brain. This may be due to the species difference. Indeed, the soma of GVB-containing neurons in the human hippocampus is larger than that of their mouse counterparts, and the size of GVB cores is proportionally different. In addition, experimental GVBs may present a different stage in GVB development than GVBs that are observed in the context of GVD in the human brain. This may relate to the difference between acute experimental induction of tau pathology and the more gradual process in the human brain. In conclusion, the GVBs in our novel models carry the protein signature and harbor the morphological properties of GVBs in the human brain.

### GVBs are proteolytically active lysosomal structures that accumulate endocytic and specific cytosolic cargo

The late endosomal marker CHMP2B consistently labels GVBs in the human brain and tau Tg mouse models [45, 78]. In the present study, GVBs in vitro are also positive for CHMP2B, but not for the early endosomal marker EEA1. CHMP2B is a subunit of the endosomal sorting complex required for transport III (ESCRT-III), which functions in the invagination and scission of intraluminal vesicles in late endosomes (for review see Ref. [59]). Recently, a subset of CK1δ-labeled GVBs in the human hippocampus was also found immunoreactive for VPS4a, the CHMP2B-interacting protein that is recruited during the final membrane scission step [45]. Our ultrastructural analysis demonstrated the presence of vesicular structures in the vacuole of GVBs, which may represent intraluminal vesicles that typically occur in late endosomes. Furthermore, the presence of the bulk endocytic cargo DQ-BSA in GVBs demonstrates that GVBs are connected to the endocytic flux. Taken together, GVBs share key characteristics with late endocytic compartments.

In addition, the accumulation of cytosolic (CK1δ, CK1ɛ and peIF2α) and ER transmembrane proteins (pPERK and pIRE1α) in tau-induced GVBs indicates that autophagic processes contribute to the content of GVBs. Selective autophagic pathways exist that target specific cargo for degradation, but the majority of autophagic processes involve non-selective sequestration of cargo by a double autophagic membrane (macroautophagy) (for review see Ref. [21]). The ER is one of the major sources of the autophagic membrane [40], which could explain the presence of pPERK and pIRE1α in GVBs. However, the localization of the activated UPR markers pPERK, pIRE1α and peIF2α in the GVB core rather than in the membrane, makes it more likely that they end up in GVBs via selective targeting of parts of the ER for degradation (ER-phagy) [26].

Autophagosomes are double-membraned structures at the early stage of the autophagic pathway that are positive for LC3 [21, 34]. GVBs have been postulated to represent a type of autophagosome based on one study suggesting the presence of a double membrane surrounding the GVB core and vacuole [51]. This contrasts with the majority of ultrastructural descriptions of GVBs in the human brain that do not report a double membrane, including studies using CK1δ and CK1ɛ immunopositivity for the GVB core [23, 39]. In our in vitro model and in the human AD brain [20], GVBs are LC3-negative. Accordingly, we show that the CK1δ-immunopositive core is delineated by a single membrane using TEM. Therefore, we conclude that GVBs cannot be classified as autophagosomes.

**Fig. 9:**
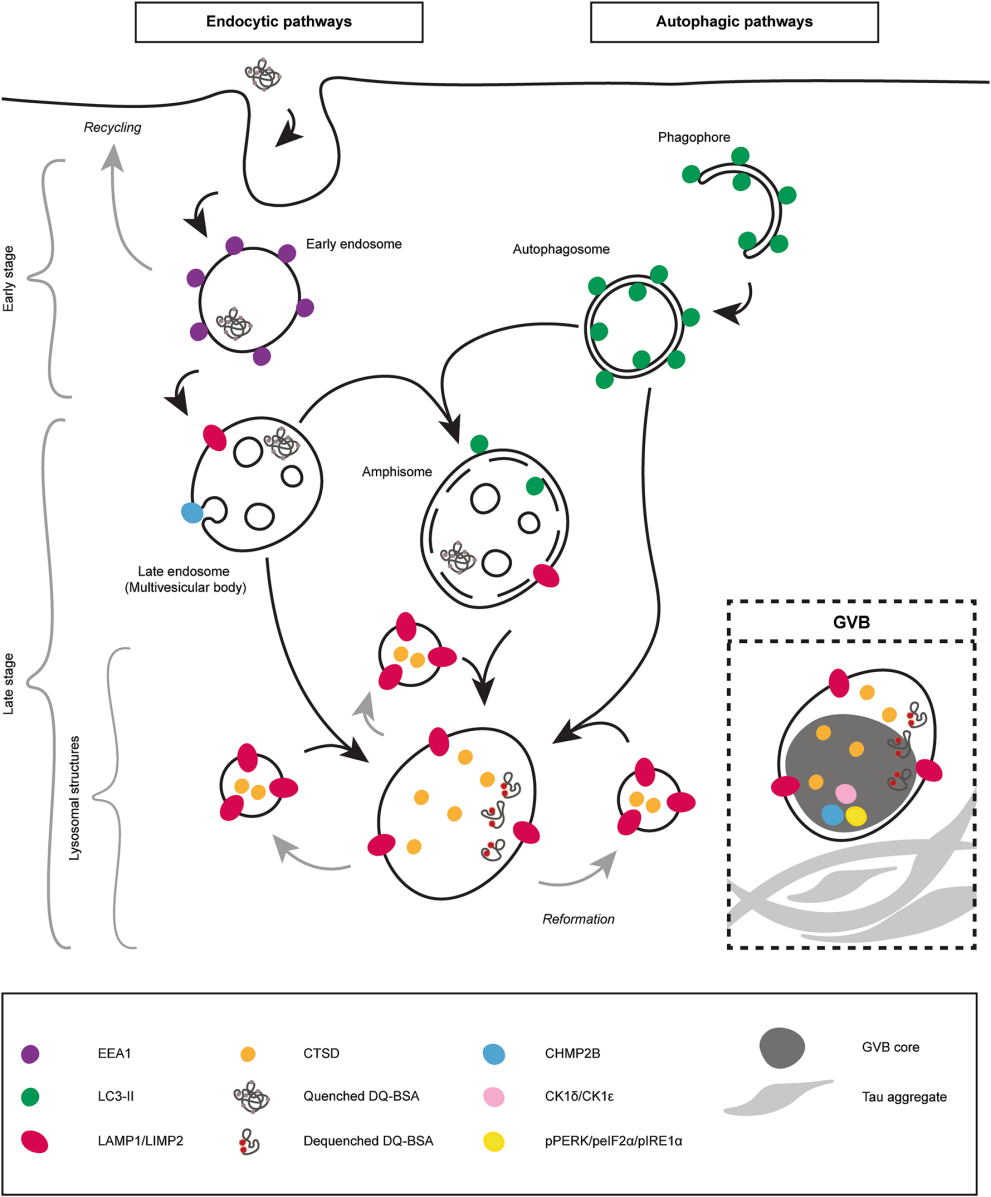
GVBs are lysosomal structures that contain accumulated endocytic and autophagic cargo. Schematic representation of the autophagic and endocytic pathways and in the inset the summary of the characterization of GVBs induced by seeded tau pathology based on the results from the novel models presented in this study. The localization of the molecular markers used to distinguish different endocytic and autophagic intermediates and to detect GVBs are indicated. Early stage endocytic and autophagic organelles comprise early endosomes, phagophores and autophagosomes. Late stage endocytic and autophagic organelles include late endosomes (or multivesicular bodies), amphisomes (resulting from the fusion of autophagosomes with late endosomes) and lysosomal structures (the fusion of late endosomes, amphisomes or autophagosomes with lysosomes results in the formation of endo- or autolysosomes) [7, 21, 36]. Note that in addition to the macroautophagic pathway depicted here, microautophagy, chaperone-mediated autophagy and other selective autophagy pathways also target cytosolic content for degradation [21]. Our data identify GVBs as proteolytically active lysosomal structures that can be distinguished from physiological lysosomal structures by a dense core that contains (specific) accumulated cargo. See text for further detail

Moreover, tau-induced GVBs contain CTSD and their membranes are positive for the transmembrane proteins LAMP1 and LIMP2, all markers of late autophagic and endocytic organelles. These data are consistent with findings in the human AD brain [20]. In addition to CTSD, GVBs contain the proteolysis reporter DQ-BSA, indicating proteolytic activity. Taken together, the presence of late endocytic and autophagic as well as proteolytic markers, identifies GVBs as lysosomal structures at the convergence of the endocytic and autophagic pathway (Fig. 9).

The typical distinguishing feature of the GVB-type lysosomal structure is their dense core in which endocytic, ER and cytosolic proteins accumulate. Our data indicate selectivity in the accumulation of cytosolic proteins in GVBs. Previous EM studies in the human brain did not detect filamentous material inside GVBs [32, 51] and analyses of the presence of tau epitopes in GVBs in human post-mortem brain have generated contradictory results (for review see Ref. [37]). We demonstrate that neither aggregated or diffuse GFP-tagged tau nor the tau seeds accumulate in GVBs and no fibrillar structures were detected in GVBs by TEM. Occasional AT100 immunoreactivity was observed in GVBs. It has been suggested that the high abundance of phospho-epitopes in human GVBs may be explained by the presence of processed protein fragments. Possibly, monomeric tau-GFP is not present in GVBs because it is efficiently sequestered in the tau aggregates that are too large to be contained in GVBs or other lysosomal structures. Alternatively, tau may be efficiently degraded in GVBs. Taken together, the current study indicates that tau does not preferentially accumulate in GVBs, although the presence of low levels of tau or tau fragments cannot be excluded.

In contrast to tau-GFP or GFP alone, CK1δ-GFP accumulates in GVBs. This finding indicates selective targeting of cytosolic cargo to GVBs. Selective targeting of specific proteins to late endosomes and lysosomal structures by microautophagy and chaperone-mediated autophagy, respectively, requires a KFERQ consensus sequence [21, 57, 70]. However, CK1δ does not contain this motif. Other selective pathways make use of specific autophagy receptors. The CK1δ yeast homologue Hrr25 has been shown to regulate selective autophagy by phosphorylation of different autophagy receptors [47, 53, 65, 82]. If this function is conserved in mammalian CK1δ, its direct involvement in the selective targeting of cargo could explain its localization to GVBs.

### GVB formation: a neuron-selective lysosomal response to tau pathology

In the human AD brain, the hippocampus is more severely affected by GVD than the cortex, following the Braak staging for NFTs. Introduction of tau pathology in cultured hippocampal or cortical neurons demonstrates that the GVB load is similarly correlated with tau load in these neuronal subtypes. The striatum is relatively spared from GVD in the AD brain, but forced introduction of tau pathology in striatal neurons in vitro is accompanied by GVB formation. These results are in line with a model in which GVBs are a neuronal response to tau pathology that is independent of the brain region from which they originate. Overall, our data suggest that the regional preference for the formation of GVBs is a consequence of the regional vulnerability for tau pathology.

Whereas neurons formed GVBs in the presence of tau pathology, primary astrocytes and HEK293 cells did not. In AD, tau pathology mainly manifests in neurons, but in other tauopathies glial tau inclusions are a more prominent part of the pathology. This cell type-specific appearance of tau pathology is reproduced using patient-derived tau seeds to induce tau pathology in mice [12, 49]. GVBs have been reported in glial cells with tau pathology in patients with Pick’s disease, familial FTD and a CBD/PSP-like tauopathy [50, 69], but not in cases with PSP, parkinsonism dementia complex of Guam, pallido-ponto-nigral degeneration patients [60] and aging-related tau astrogliopathy (ARTAG) [45]. Here, we show that GVBs are abundantly present in tau-positive neurons, whereas they are only occasionally observed in tufted astrocytes in ALZ17 mice injected with human PSP brain homogenate. This is in agreement with the observation of GVBs in glia in the human brain and indicates that the occurrence of GVBs is neuron-selective, but not neuron-exclusive. Data from our in vitro model demonstrate that the neuron-selective occurrence of GVBs in the human brain is not caused by differential susceptibility of glia and neurons to tau pathology, but rather by a cell type-specific response to this.

Neurons are highly specialized post-mitotic cells, which makes them a priori more dependent on a functional lysosomal system than glia cells of which at least subpopulations retain proliferative capacity [11]. The extreme neuronal polarization requires long distance trafficking of organelles and cargo, in which the microtubule-binding protein tau plays an important role that could be disturbed by recruitment of tau to aggregates [73]. This may underlie the correlation between GVB-type lysosomal structures and tau pathology and their selective occurrence in neurons. Indeed, pathological tau leads to the accumulation of organelles and cargo in the neuronal soma [24, 63], where physiological lysosomes also reside [18, 79] and GVBs appear. Acute disruption of the microtubule network by vinblastine does not induce GVB formation, suggesting that transport block per se is not sufficient to induce GVBs.

In addition to loss of tau function, tau aggregation may disturb organelle trafficking and other cellular processes by steric hindrance [44]. GVBs emerge at the convergence of the endocytic and autophagic pathways. The proteolytic capacity is apparently insufficient to efficiently degrade the GVB core. Tau pathology presents a major disturbance of proteostasis and thereby likely impinges an increased demand on the lysosomal system to degrade pathological tau species as well as other proteins and cellular structures affected by the pathology. Overloading of the lysosomal system may lead to the accumulation of undigested cargo in GVB-type lysosomal structures. The reformation of lysosomes and thereby restoration of lysosomal homeostasis is disturbed if degradation is impaired [56, 71]. Hence, GVBs may specifically emerge during stress in the lysosomal system. An important response to lysosomal stress is mediated by the activation of the transcription factor EB (TFEB), a master regulator of lysosomal biogenesis (for review see Refs. [2, 14]). Increased expression of TFEB and TFEB targets has been demonstrated in the AD hippocampus [5]. Interestingly, TFEB overexpression in tau Tg mice induces lysosomal clearance of pathological tau, resulting in reduction of tau aggregates and amelioration of neurodegeneration [54, 72]. Future studies should address whether (dysregulation of) the TFEB response by the chronic proteostatic disturbance presented by tau aggregation is involved in GVB formation. The characterization of GVBs and the novel models that we present here, provide new opportunities to further investigate the functional involvement of GVBs in tau pathogenesis.

## Electronic supplementary material

Below is the link to the electronic supplementary material.

Supplementary material 1 (PDF 147657 kb)

**Online Resource 2: Tau-induced single core GVBs visualized by STED super-resolution microscopy.** Movie of a Z-stack obtained by STED microscopy of primary neurons expressing tau-P301L-GFP (gray) treated with PFFs and immunostained with CK1δ (green) and LIMP2 (red). Shown are tau aggregates, two single core GVBs (arrowheads) and non-GVB LIMP2-positive structures (AVI 417 kb)
