# Supplementary material for: Granulovacuolar degeneration bodies are neuron-selective lysosomal structures induced by intracellular tau pathology
Source: Acta Neuropathol. 2019 Aug 27;138(6):943–70. doi: 10.1007/s00401-019-02046-4 (PMC6851499; doi:10.1007/s00401-019-02046-4)
Supplement: Supplementary file 1 — Supplementary material 1 (PDF 147657 kb) [file 401_2019_2046_MOESM1_ESM.pdf]

*Granulovacuolar degeneration bodies are neuron-selective lysosomal structures induced by intracellular tau pathology*

Vera I. Wiersma[1,2], Anna Maria van Ziel[1,2], Sonia Vazquez-Sanchez[1], Anna Nölle[3], Ernesto Berenjeno-Correa[1], Anna Bonaterra-Pastra[1], Florence Clavaguera[6], Markus Tolnay[7], René J.P. Musters[4], Jan R.T. van Weering[2], Matthijs Verhage[1,2], Jeroen J.M. Hoozemans[3] and Wiep Scheper[1,2,5*]

[1]Department of Functional Genomics, Center for Neurogenomics and Cognitive Research, Vrije Universiteit (VU), Amsterdam, The Netherlands. [2]Department of Clinical Genetics, [3]Department of Pathology, [4]Department of Physiology, [5]Alzheimer Center, Amsterdam University Medical Centers location VUmc, Amsterdam, The Netherlands. [6]Institut du Cerveau et de la Moelle épinière, INSERM U1127, CNRS UMR7225, Sorbonne Universités, Hôpital Pitié-Salpêtrière, Paris, France. [7]University Hospital Basel, Institute of Medical Genetics and Pathology, CH-4031 Basel, Switzerland

*Correspondence to

W. Scheper
VU Faculty of Science
Center for Neurogenomics and Cognitive Research
Department of Functional Genomics
De Boelelaan 1085
1081 HV Amsterdam
The Netherlands

E-mail: w.scheper@amsterdamumc.nl
Phone: +31-20-5982771

**Acta Neuropathologica Online Resource 1: Supplementary Figures and Supplementary Tables**

# Supplementary Figure 1

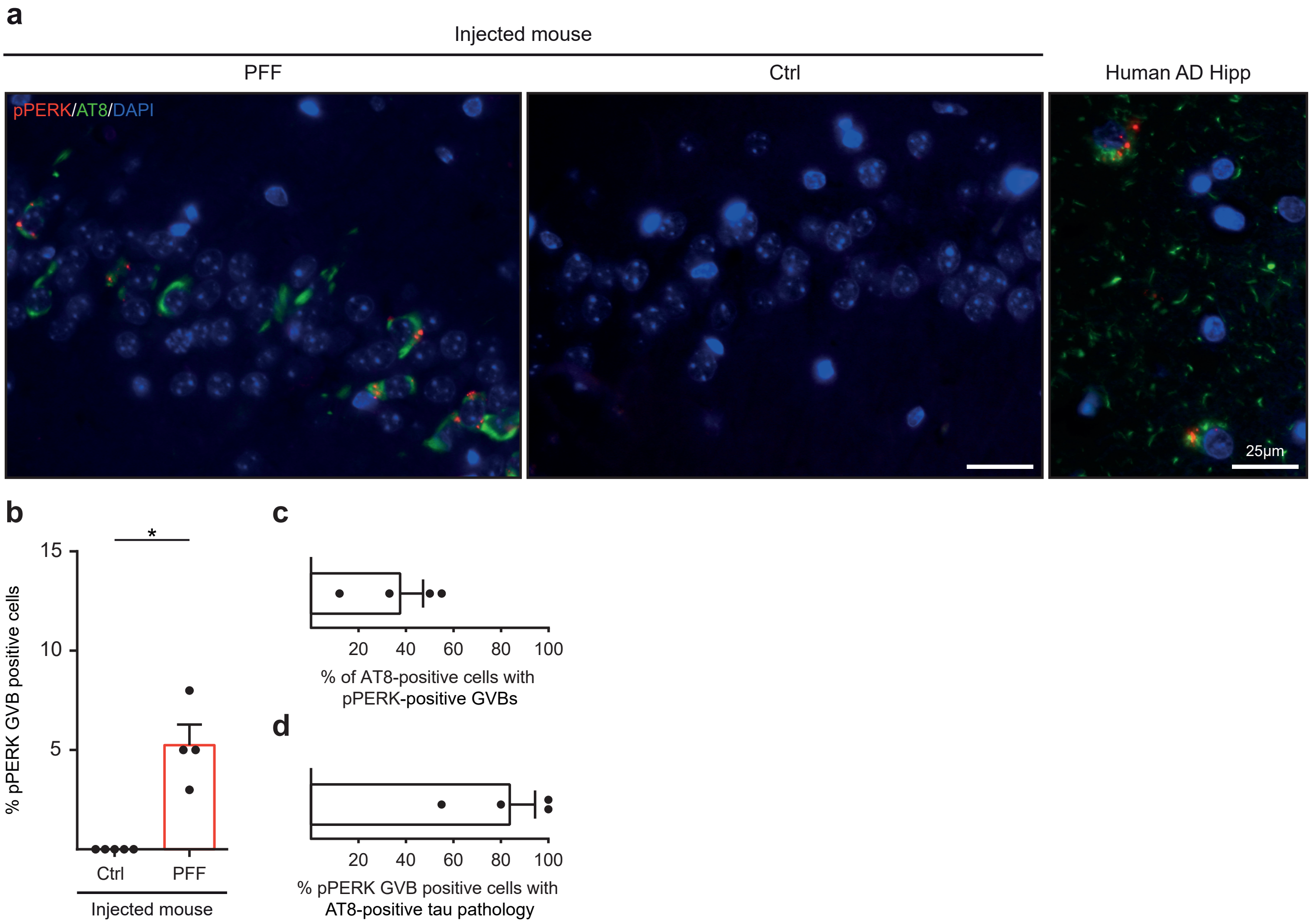


**Supplementary Fig. 1** Seeding of tau pathology using PFFs induces GVBs *in vivo*. **a-c** Immunofluorescence was performed on hippocampal sections of tau P301L Tg mice injected with K18 tau P301L seeds (PFF, $N = 5$) or control buffer (Ctrl, $N = 5$). **a** Representative epifluorescence images of immunostaining using AT8 (green) and the GVB marker pPERK (red). Note that the AT8 epitope is absent from K18 PFFs. **b-d** Quantification of **a** showing the percentage of pyramidal neurons positive for pPERK in the hippocampal CA1, CA2 and CA3 area of Ctrl- or PFF-injected mice (**b**). * $p < 0.05$, one sample *t*-test. **c** Quantification of the percentage of AT8-immunoreactive neurons that contains pPERK-labeled GVBs and **d** of neurons with pPERK-positive GVBs that is also positive for AT8 in the hippocampus of PFF-injected mice. Bars indicate the mean + SEM. Data points represent individual animals. See Supplementary Table 2 for an overview of the number of cells analyzed

# Supplementary Figure 2

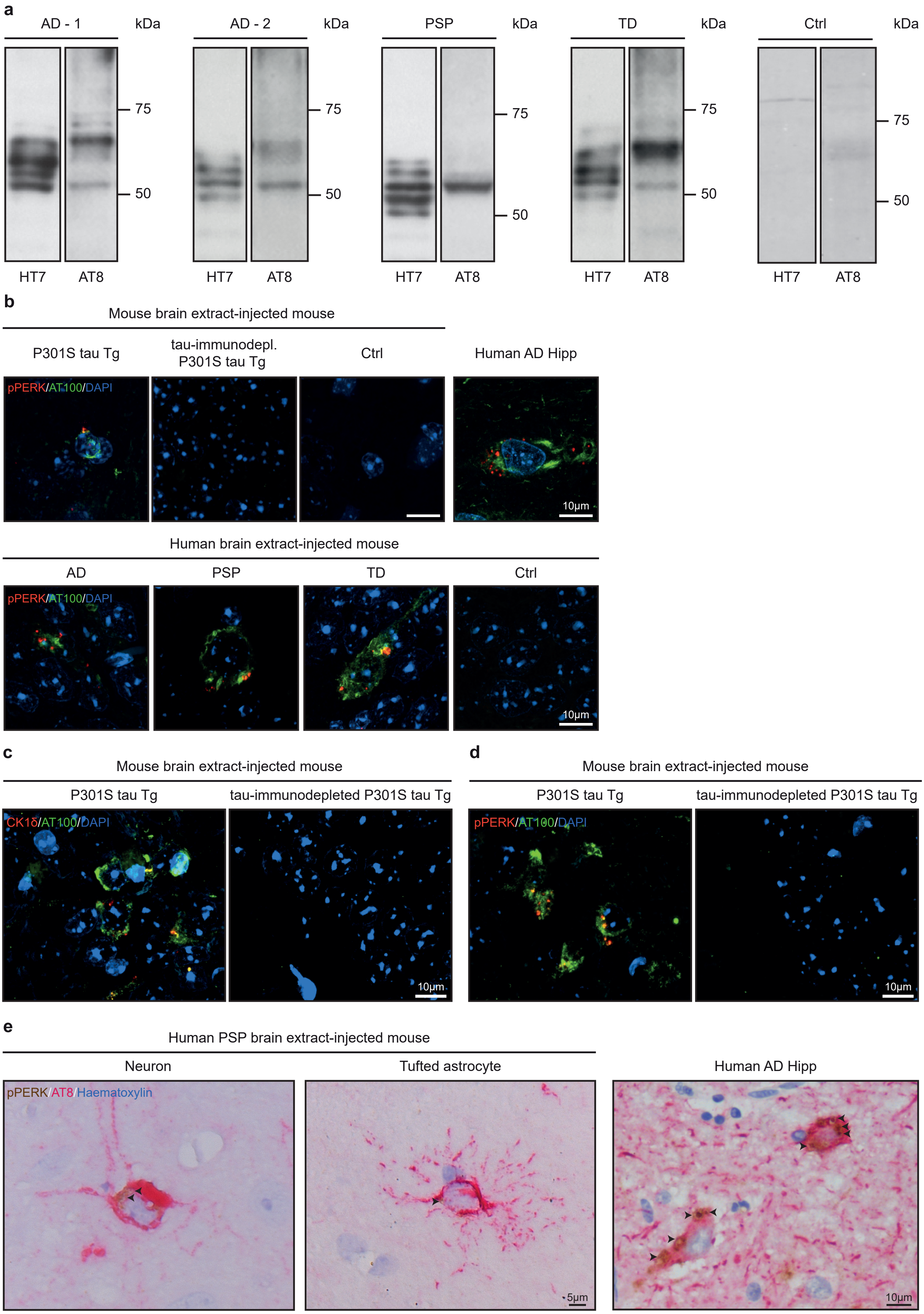

**Supplementary Fig. 2** Seeding of tau pathology using brain homogenate induces GVBs *in vivo*. ALZ17 mice were injected with extract from mouse brain (tau P301S Tg (*N* = 6), tau P301S Tg immunodepleted of tau (tau-immunodepl., *N* = 4), non-Tg C57BL/6 mice (Ctrl, *N* = 1)) or human post-mortem brain (AD (*N* = 6), PSP (*N* = 3), TD (*N* = 4) patients or controls (Ctrl, *N* = 4)). **a** Western blots of human brain lysates used for injection. See Supplementary Table 1 for patient details. Tau was visualized on the blot using antibodies for total tau (HT7) and phosphorylated tau (AT8). Western blots for tau on brain lysates from non-Tg C57BL/6 and tau P301S Tg mice (before and after successfull immunodepletion) used for injection were previously published in [1]. **b** Representative confocal images of immunofluorescence with AT100 (green) and the GVB marker pPERK (red) in the injected mice. Human AD hippocampus (Human AD Hipp) is included for reference. **c,d** Representative confocal images of immunofluorescence with AT100 (green) and CK1δ (**c**) or pPERK (**d**) (red) in mice injected with tau P301S Tg brain lysate before or after immunodepletion of tau. Zooms of these conditions are shown in Fig. 1h and Supplementary Fig. 2b, respectively. Cell nuclei are stained with DAPI (blue) in all images. **e** Representative images of double immunohistochemistry with AT8 and pPERK antibodies in mice injected with human PSP brain lysate. Immunohistochemical detection is visualized by DAB and LPR labelling pPERK (brown) and AT8 (red), respectively. Arrowheads point to examples of GVBs. Cell nuclei are counterstained with haematoxylin (blue). Human AD hippocampus (Human AD Hipp) is included for reference

# Supplementary Figure 3

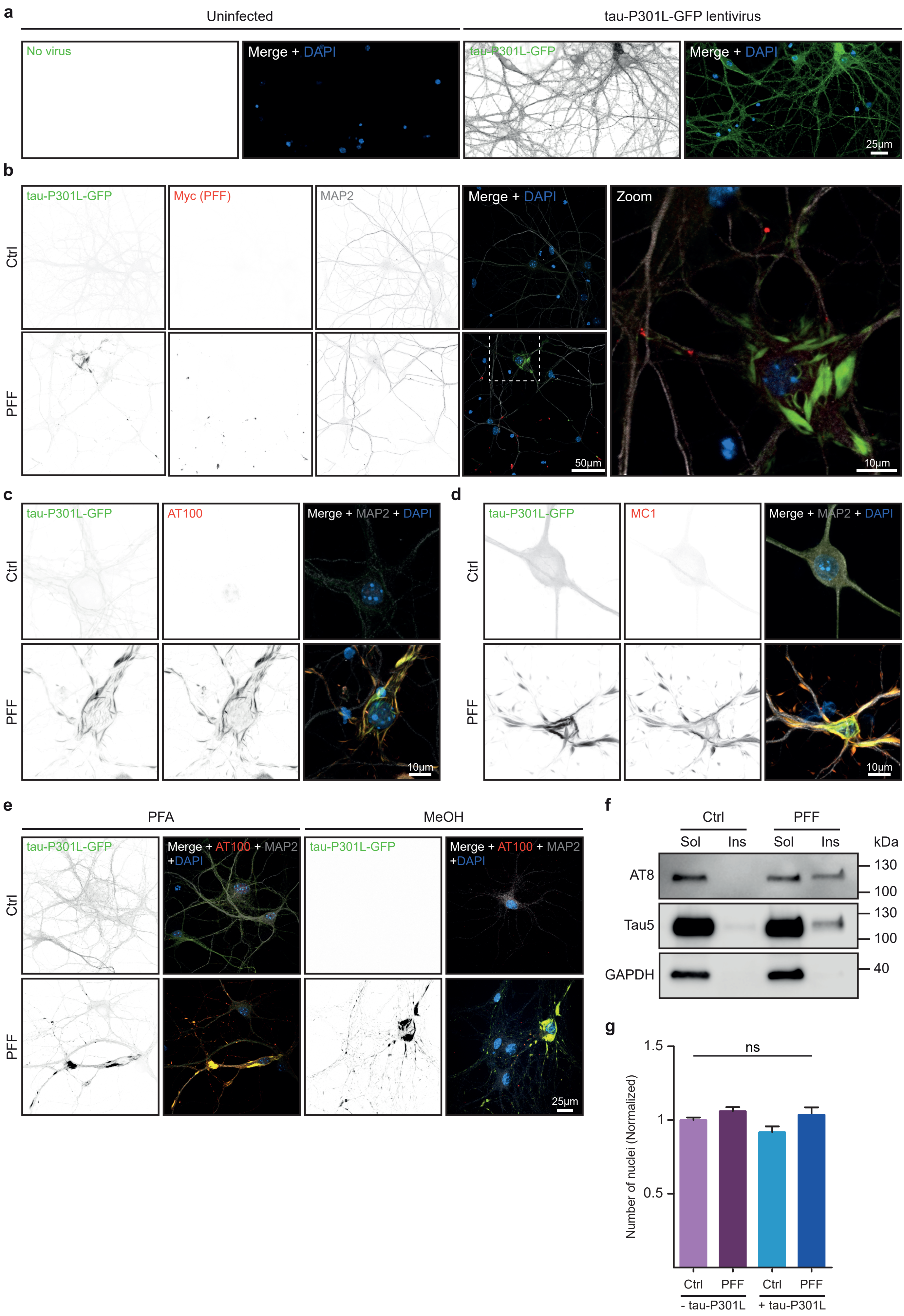

**Supplementary Fig. 3** Tau seeding induces intracellular tau pathology in primary mouse neurons. Primary mouse hippocampal neurons expressing human tau P301L were treated with K18 tau P301L seeds (PFF) or control buffer (Ctrl) at DIV 7 and fixed at DIV 18. Untagged, GFP- and mCherry-tagged human tau P301L lentiviral constructs were used in experiments. The validation shown here for tau-P301L-GFP applies to all lentiviral tau constructs. Individual fluorescence signals are shown separately in grayscale and in color in the merge. Tau-P301L-GFP is always shown in green. Cell nuclei are stained with DAPI (blue) in all images. **a** Representative confocal images of neurons with or without lentivirus-mediated expression of tau-P301L-GFP. Images were acquired with optimal settings for non-aggregated tau. **b** Representative confocal images and zoom of immunolabeling for the Myc-tag coupled to PFFs (red) and the neuron-specific dendritic marker MAP2 (gray). Zoomed area is indicated in the merged image. Zoom shows a single focal plane, whereas the other images are maximum intensity projections of Z-stacks. The overlap between the Myc and MAP2 signal in the single focal plane zoom indicates PFF internalization. **c, d** Representative confocal images of immunolabeling with AT100 (**c**) or MC1 (**d**) (red) and MAP2 (gray). Note that the AT100 and MC1 epitopes are absent from K18 PFFs. **e** Representative confocal images of neurons fixed with PFA or with MeOH to remove soluble tau. Immunolabeling with AT100 (red) and MAP2 (gray) is shown. **f** Representative Western blot of the Triton X-100 soluble (Sol) and insoluble (Ins) fractions of lysates from tau-P301L-GFP expressing neuronal cultures. Membranes were immunoblotted with AT8 and total tau (Tau5). GAPDH served as a loading and fractionation control. *N* = 2 independent experiments. **g** Neurons with (+) or without (-) tau-P301L expression were treated with PFFs or Ctrl. Quantification of the number of nuclei based on the DAPI signal in the conditions shown in Fig. 3a. Image acquisition and analysis were automated. Bars indicate the mean of the observations + SEM. *N* = 3 independent experiments, *n* = 16 observations per condition, on average 6101 nuclei per observation. ns = not significant, Kruskall-Wallis test followed by Dunn’s multiple comparison test

# Supplementary Figure 4

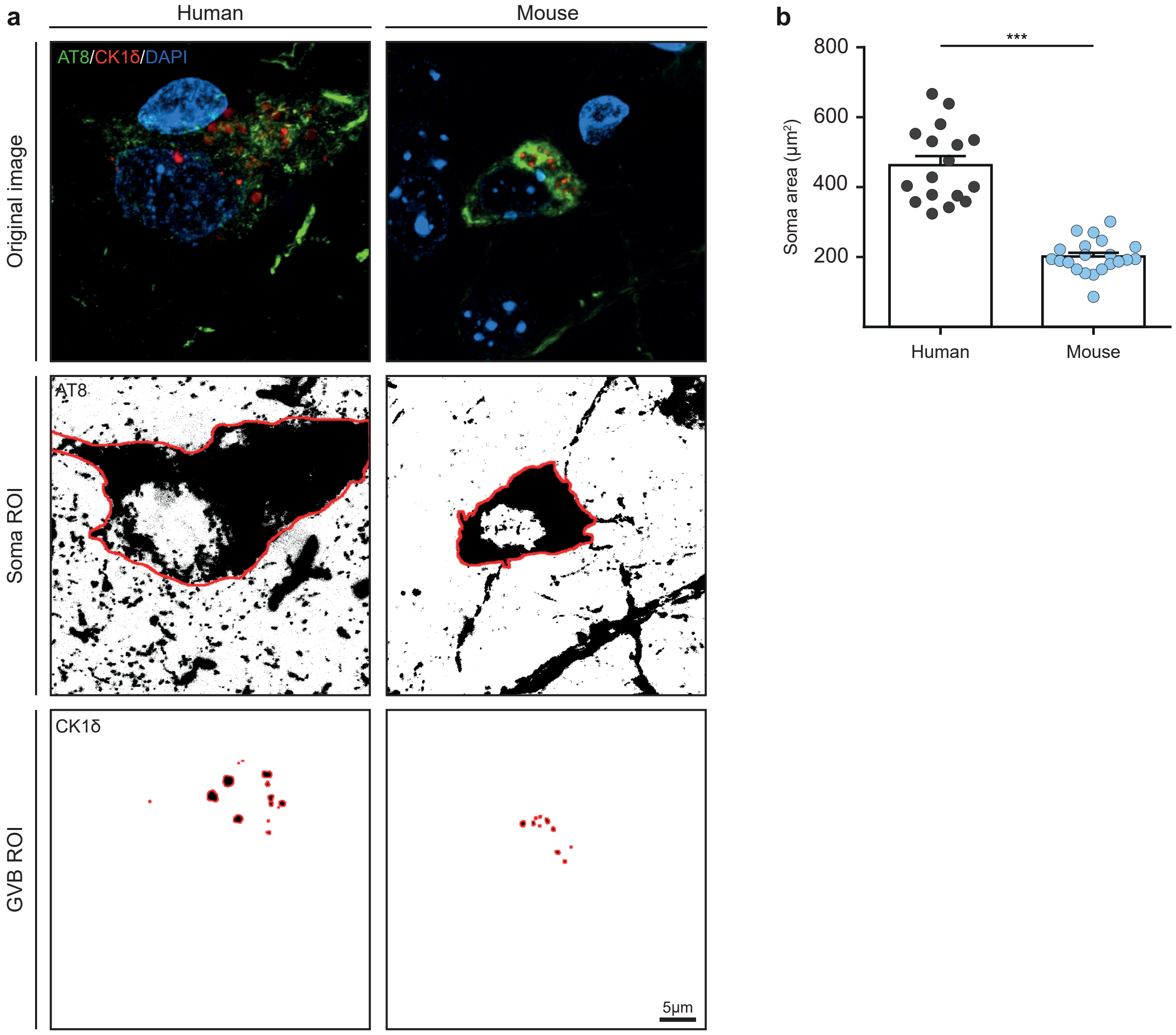


**Supplementary Fig. 4** Soma size of GVB-containing neurons is smaller in mouse than in human hippocampus. Immunofluorescence was performed on human AD hippocampus (Human) and PFF-injected tau P301L Tg mouse hippocampal sections (Mouse). **a** Representative confocal images and derived regions of interest (ROI) of immunostaining using AT8 (green) and the GVB marker CK1δ (red). Cell nuclei are stained with DAPI (blue). Soma size was determined based on the AT8 signal in the maximum intensity projected Z-stack. GVB size was determined based on the CK1δ signal in a single focal plane and quantification is shown in Fig. 2g. **b** Quantification of the area of the soma of GVB-positive hippocampal neurons using the AT8-based ROI in the conditions shown in **a**. Datapoints represent individual neurons. Bars indicate the mean + SEM. $N \geq 4$ independent experiments, $n$ = 17 and 21 neurons analyzed for Human and Mouse, respectively. *** $p < 0.001$, Student's *t*-test

# Supplementary Figure 5

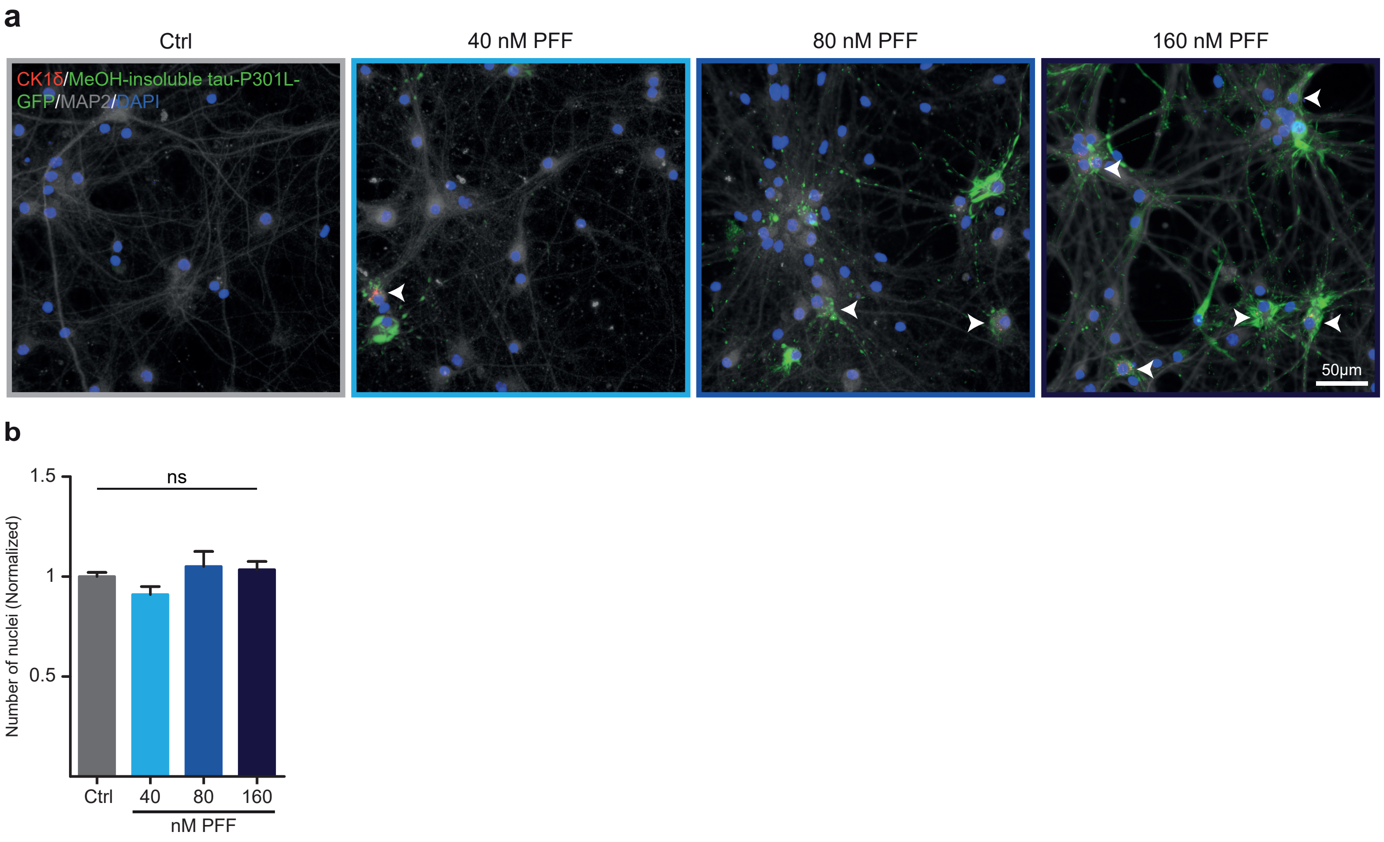


**Supplementary Fig. 5** Supplementary data accompanying the correlation of tau and GVB load. Neurons expressing tau-P301L-GFP (green) were treated with 40, 80 or 160 nM PFFs or control buffer (Ctrl). At the endpoint of the experiment soluble tau was extracted using ice-cold MeOH. Neurons were immunostained for the GVB marker CK1δ (red) and neuron-specific dendritic marker MAP2 (gray). Cell nuclei are stained with DAPI (blue). Image acquisition and analysis were automated. **a** Representative epifluorescence images of the conditions quantified in Fig. 3f are shown. Arrowheads point to examples of neurons with GVBs. **b** Quantification of the number of nuclei based on the DAPI signal in the conditions shown in **a** and in Fig. 3e. Bars indicate the mean of the observations + SEM. *N* = 3 independent experiments, *n* = 16-18 observations per condition, on average 7747 nuclei per observation. ns = not significant, Kruskall-Wallis test followed by Dunn’s multiple comparison test

# Supplementary Figure 6

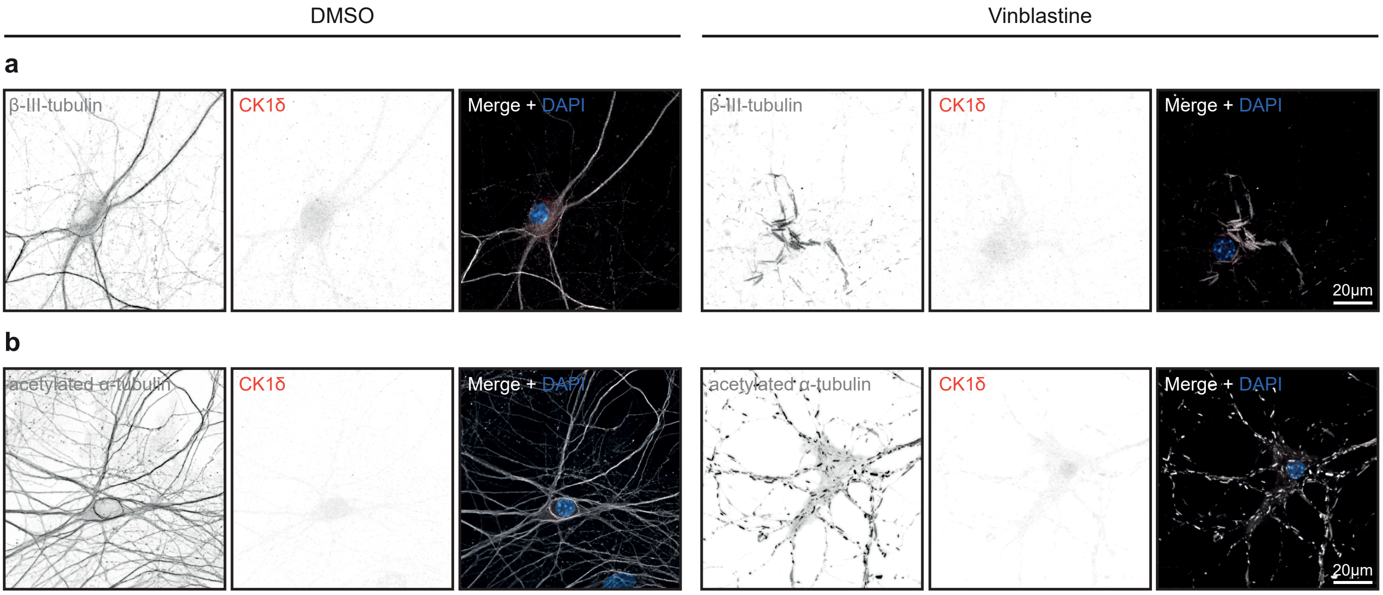


**Supplementary Fig. 6** Microtubule disruption does not induce GVBs. Primary mouse hippocampal neurons (DIV 14) were treated with 25 µM Vinblastine or DMSO for 24 hours. Cells were immunolabeled using antibodies against the GVB marker CK1δ and β-III-tubulin (**a**) or acetylated α-tubulin (**b**) to demonstrate disruption of the microtubule network. Individual fluorescence signals are shown separately in grayscale and in color in the merge: CK1δ in red and β-III-tubulin or acetylated α-tubulin in gray. Cell nuclei are stained with DAPI (blue) in all images. Whole coverslips with neurons (plating density: 40K) of *N* = 3 independent experiments were analyzed for the presence of GVBs

# Supplementary Figure 7

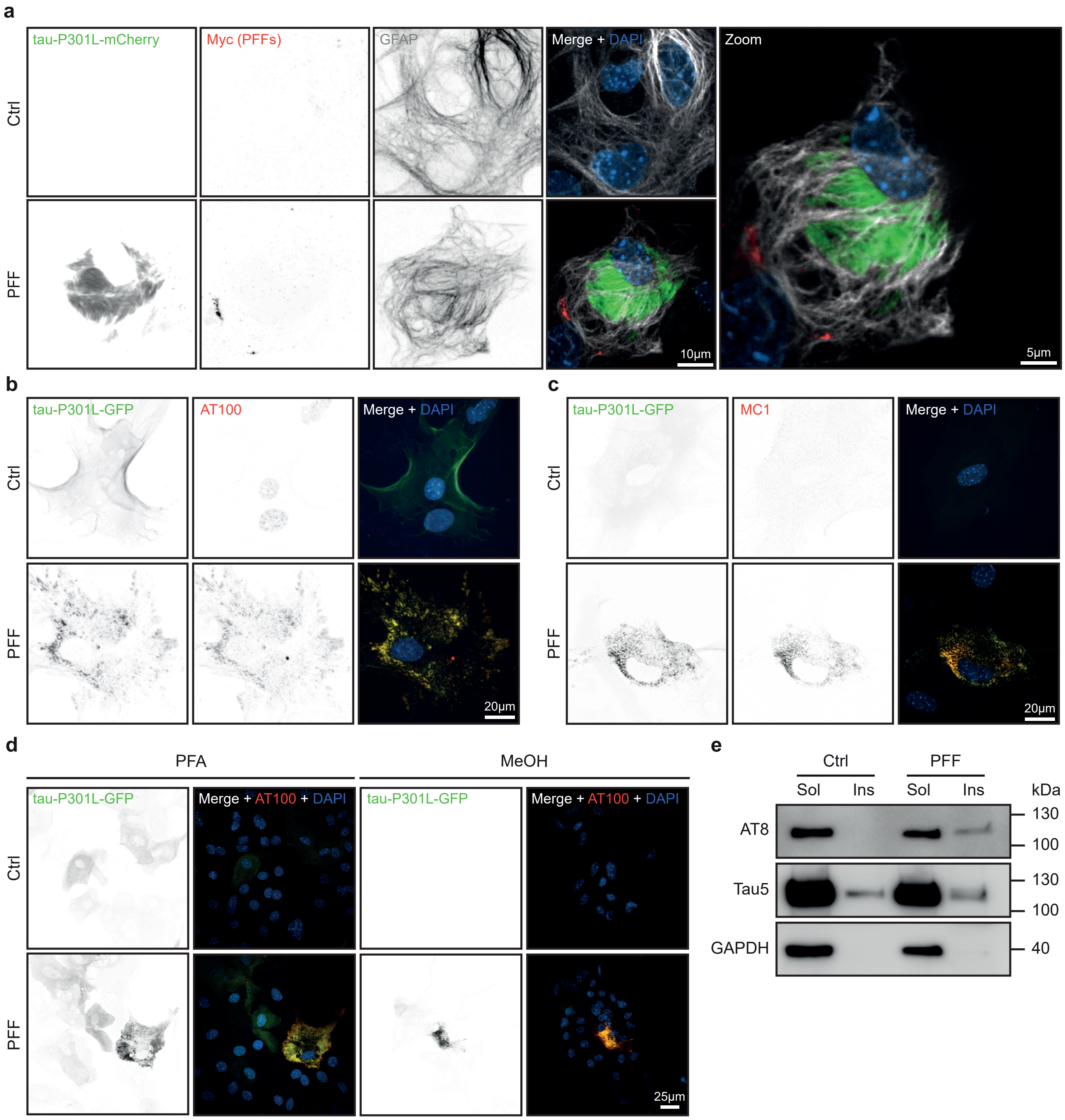


**Supplementary Fig. 7** Tau seeding induces intracellular tau pathology in primary mouse astrocytes. Primary mouse astrocytes expressing human tau P301L were treated with K18 tau P301L seeds (PFF) or control buffer (Ctrl) 7 days after medium change to NB+ and fixed 11 days later. GFP- and mCherry-tagged human tau P301L lentiviral constructs were used in experiments. The validation shown here applies to both lentiviral tau constructs. Individual fluorescence signals are shown separately in grayscale and in color in the merge. Tau-P301L-GFP/-mCherry is always shown in green. Cell nuclei are stained with DAPI (blue) in all images. **a** Representative confocal images and zoom of immunolabeling for the Myc-tagged PFFs (red) and the astrocytic marker GFAP (gray). The enlargement of the merged image of the PFF condition shows a single focal plane, whereas the other images are maximum intensity projections of Z-stacks. The overlap between the Myc and GFAP signal in the single focal plane zoom indicates PFF internalization. **b, c** Representative confocal images of immunolabeling with AT100 (**b**) or MC1 (**c**) in red. Note that the AT100 and MC1 epitopes are absent from K18 PFFs. **d** Representative confocal images of astrocytes fixed with PFA or with MeOH to remove soluble tau. Immunolabeling was performed using AT100 (red). **e** Representative Western blot of the Triton X-100 soluble (Sol) and insoluble (Ins) fractions of lysates from tau-P301L-GFP expressing astrocytic cultures. Membranes were immunoblotted with AT8 and total tau (Tau5). GAPDH served as a loading and fractionation control. *N* = 2 independent experiments

# Supplementary Figure 8

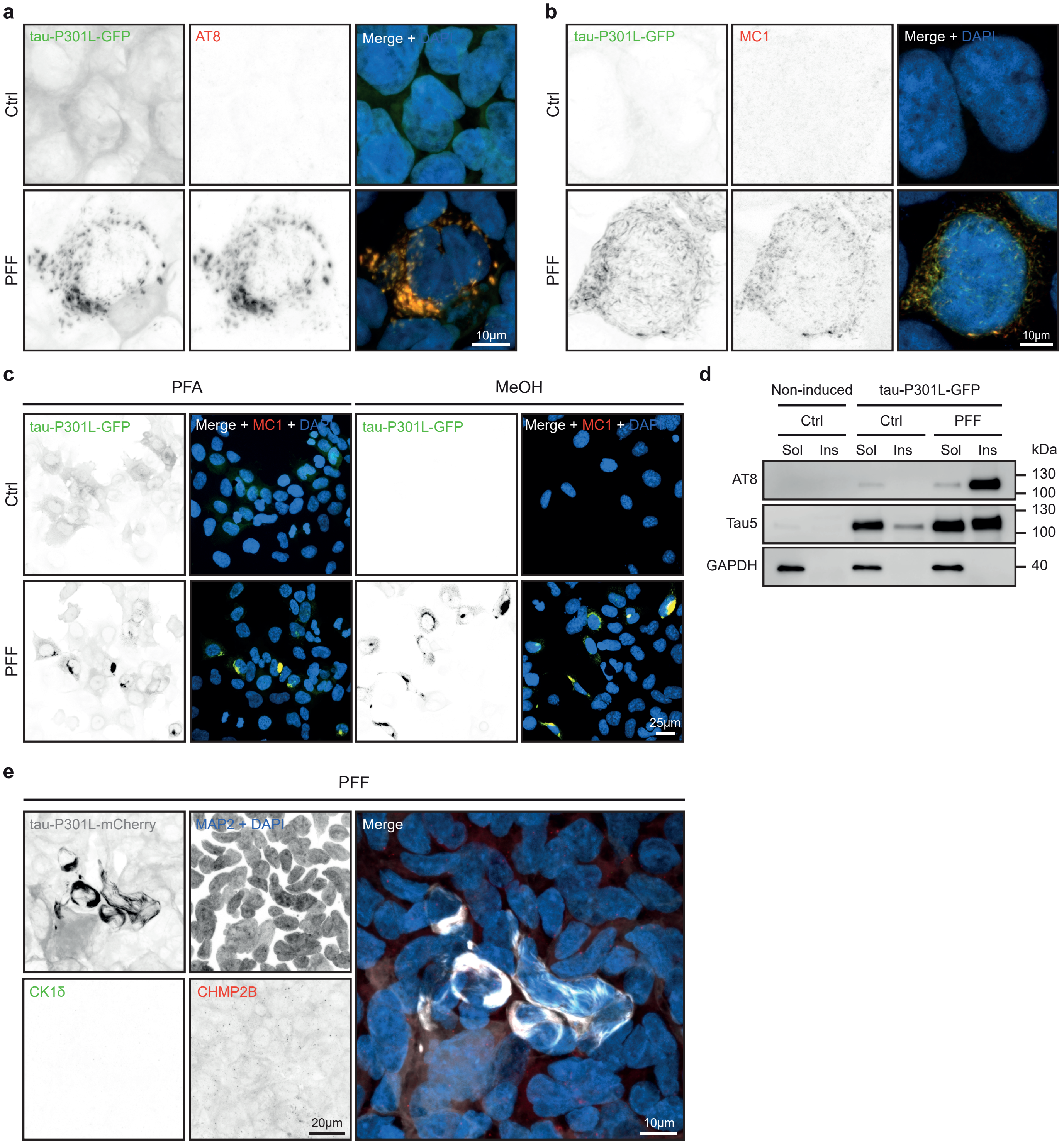


**Supplementary Fig. 8** Tau seeding induces intracellular tau pathology without detectable GVBs in HEK293 cells. **a-c** HEK293 cells with Doxycycline (Dox)-induced expression of tau-P301L-GFP were treated with K18 tau P301L seeds (PFF) or control buffer (Ctrl) 24 hours after induction of transgene expression and fixed 7 days after treatment. Individual fluorescence signals are shown separately in grayscale and in color in the merge. Tau-P301L-GFP is always shown in green. Cell nuclei are stained with DAPI (blue) in all images. **a, b** Representative confocal images of Dox-induced HEK293 cells immunolabeled with AT8 (**a**) or MC1 (**b**) (red). Note that the AT8 and MC1 epitopes are absent from K18 PFFs. **c** Representative confocal images of induced HEK293 cells fixed with PFA or with MeOH to remove soluble tau. Immunolabeling was performed using MC1 (red). **d** Representative Western blot of the Triton X-100 soluble (Sol) and insoluble (Ins) fractions of HEK293 cell lysates with or without Dox-induction of tau-P301L-GFP expression. Membranes were immunoblotted with AT8 and total tau (Tau5). GAPDH served as a loading and fractionation control. *N* = 4 independent experiments. **e** Representative confocal images of Dox-induced and PFF-treated HEK293 cells immunolabeled with the GVB markers CK1δ and CHMP2B and the neuron-specific dendritic marker MAP2. Individual fluorescence signals are shown separately in grayscale and in color in the merge: CK1δ in green, CHMP2B in red, tau-P301L-GFP in gray and MAP2 and the nuclear stain DAPI both in blue. Whole coverslips with confluent HEK293 cells of *N* = 2 independent experiments were analyzed for the presence of GVBs

# Supplementary Figure 9

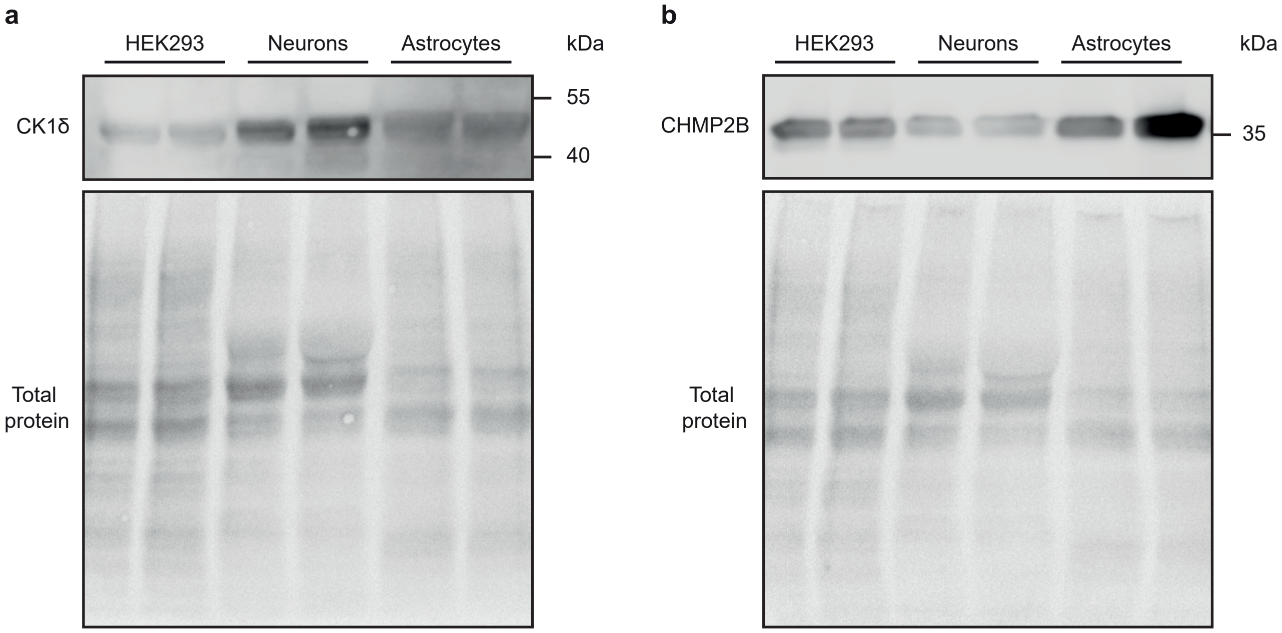


**Supplementary Fig. 9** Expression of CK1δ and CHMP2B in HEK293 cells, primary mouse neurons and astrocytes. **a, b** Representative Western blot of lysates derived from HEK293 cells (7 days in culture) expressing tau-P301L-GFP, primary mouse neurons and primary mouse astrocytes (DIV 18) immunoblotted for CK1δ (**a**) or CHMP2B (**b**). Total protein on the membrane was visualized as loading control. CK1δ: *N* = 3 independent experiments for neurons and astrocytes, *N* = 1 independent experiment for HEK293 cells. CHMP2B: *N* = 2 independent experiments for neurons and astrocytes, *N* = 1 independent experiment for HEK293 cells

# Supplementary Figure 10

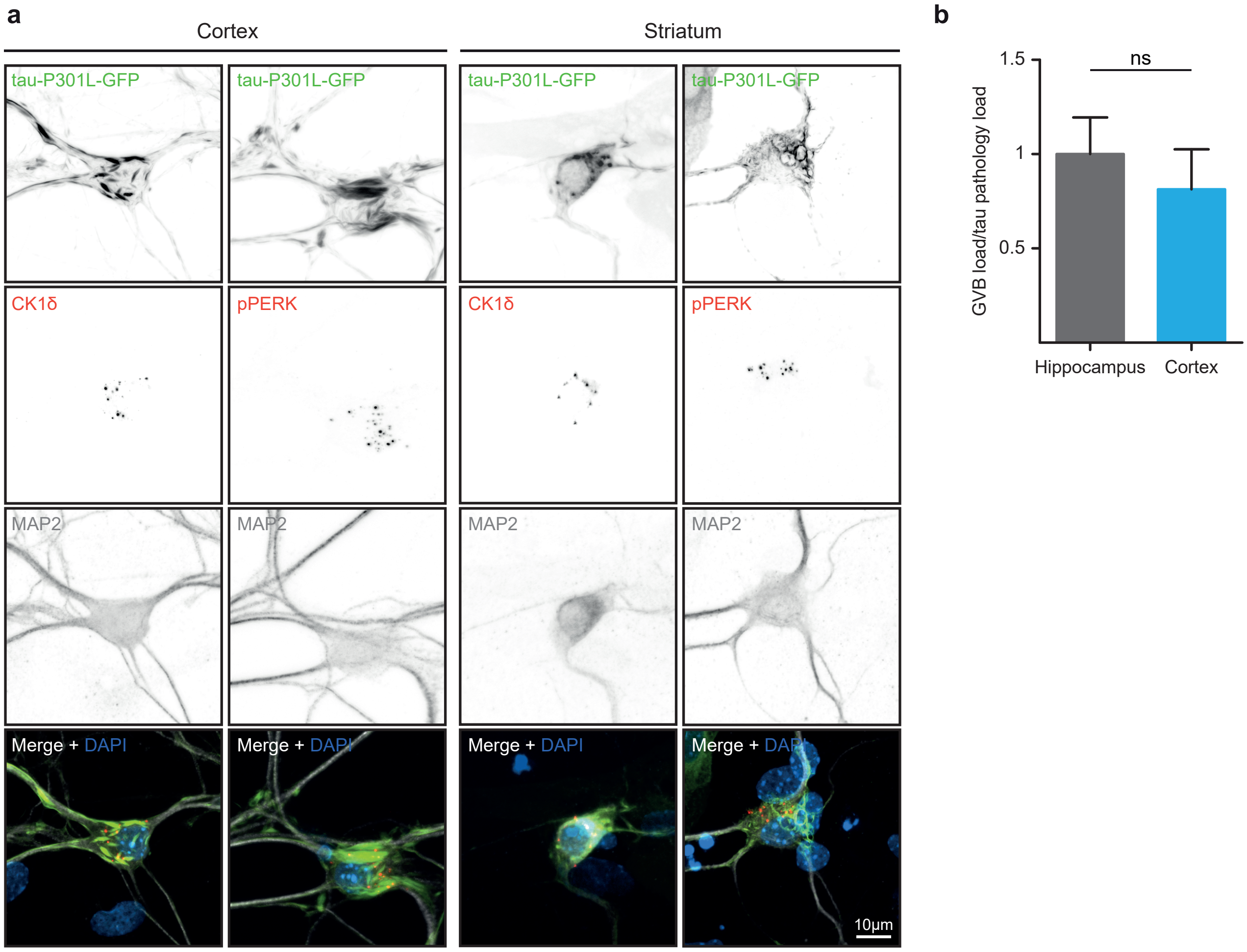


**Supplementary Fig. 10** GVBs form in cortical and striatal neurons with seeded tau pathology. Neurons cultured from cortex or stratium expressing tau-P301L-GFP (green) were treated with PFFs. **a** Representative confocal images of immunolabeling with the GVB markers CK1δ or pPERK and the neuron-specific dendritic marker MAP2 (gray). Individual fluorescence signals are shown separately in grayscale and in color in the merge: CK1δ or pPERK in red and MAP2 in gray. Cell nuclei are stained with DAPI (blue) in all images. **b** Tau pathology and GVB load were quantified as described in the Materials and Methods and the ratio GVB load/tau pathology load is shown. The average GVB load/tau pathology load ratio in hippocampal neurons was set to 1. Bars indicate the mean of the observations + SEM of 3.5 $10^4$ (hippocampus) and 3.0 $10^4$ (cortex) neurons from $n$ = 5. ns = not significant, Student's $t$-test

# Supplementary Figure 11

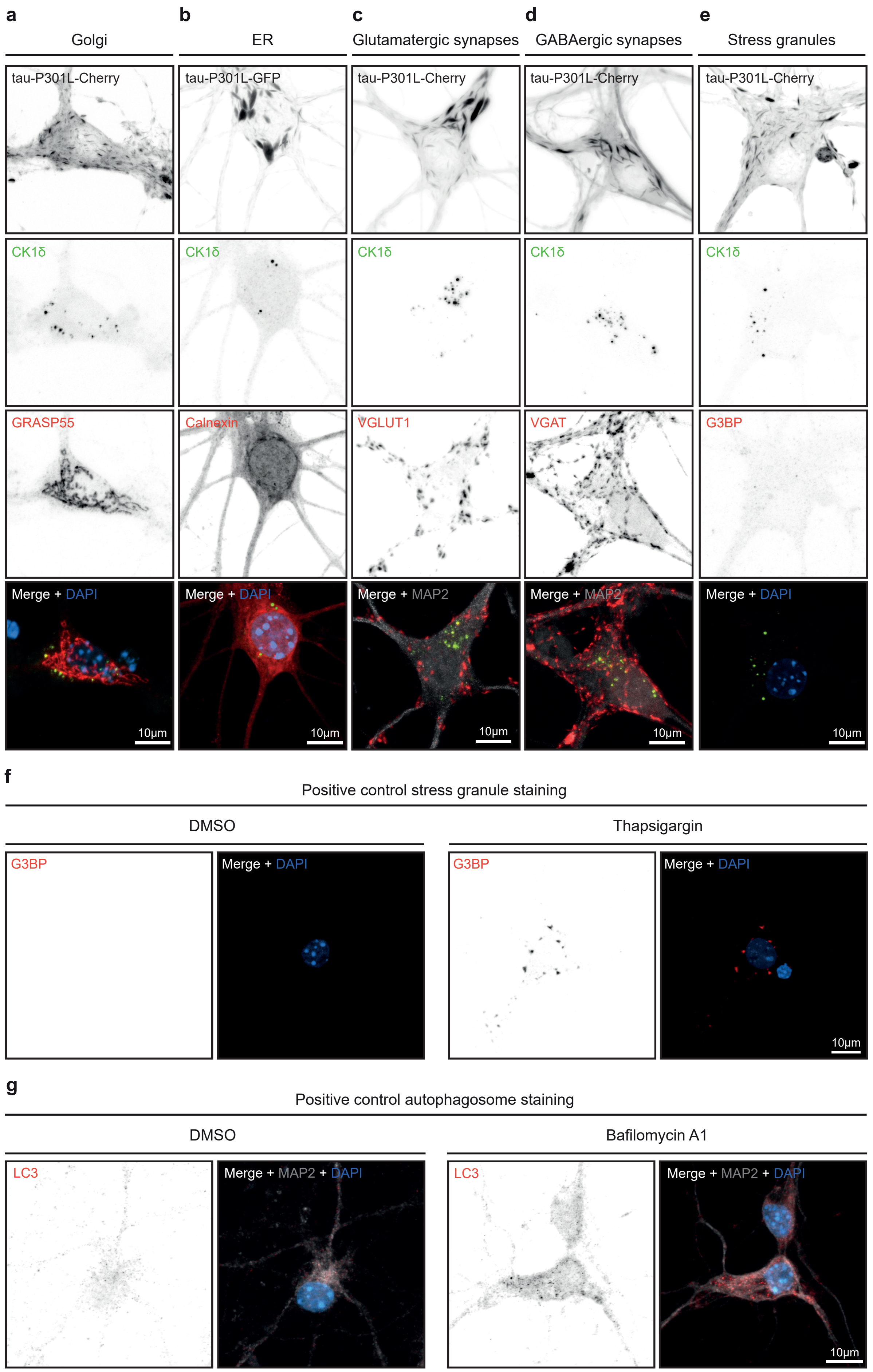

**Supplementary Fig. 11** GVBs do not overlap with non-lysosomal organelles or stress granules. **a-e** Primary hippocampal mouse neurons expressing tau P301L were treated with PFFs. Individual fluorescence signals are shown separately in grayscale and in color in the merge. Tau-P301L-GFP/-mCherry signal is not shown in the merge. Immunolabeling was performed using antibodies against the GVB marker CK1δ (green) and the Golgi marker GRASP55 (**a**), the ER marker Calnexin (**b**), the glutamatergic synapse marker VGLUT1 (**c**), the GABAergic synapse marker VGAT (**d**) and the stress granule marker G3BP (**e**) (red). See Supplementary Table 3 in Online Resource 1 for an overview of the number of cells analyzed. **f** Primary hippocampal mouse neurons (DIV 14) were treated with 10 µM Thapsigargin or DMSO for 1 hour. Cells were immunolabeled using G3BP (shown separately in grayscale and in red in the merge). This is a positive control for **e**. **g** Primary cortical mouse neurons (DIV 8) were treated with 30 nM Bafilomycin A1 or DMSO for 24 hours. Cells were immunolabeled using LC3 (shown separately in grayscale and in red in the merge). This is a positive control for Fig. 5a. Cell nuclei are stained with DAPI (blue) in **a**, **b**, **e**-**g**. In **c**, **d**, **g** the neuron-specific dendritic marker MAP2 is shown in gray

# Supplementary Figure 12

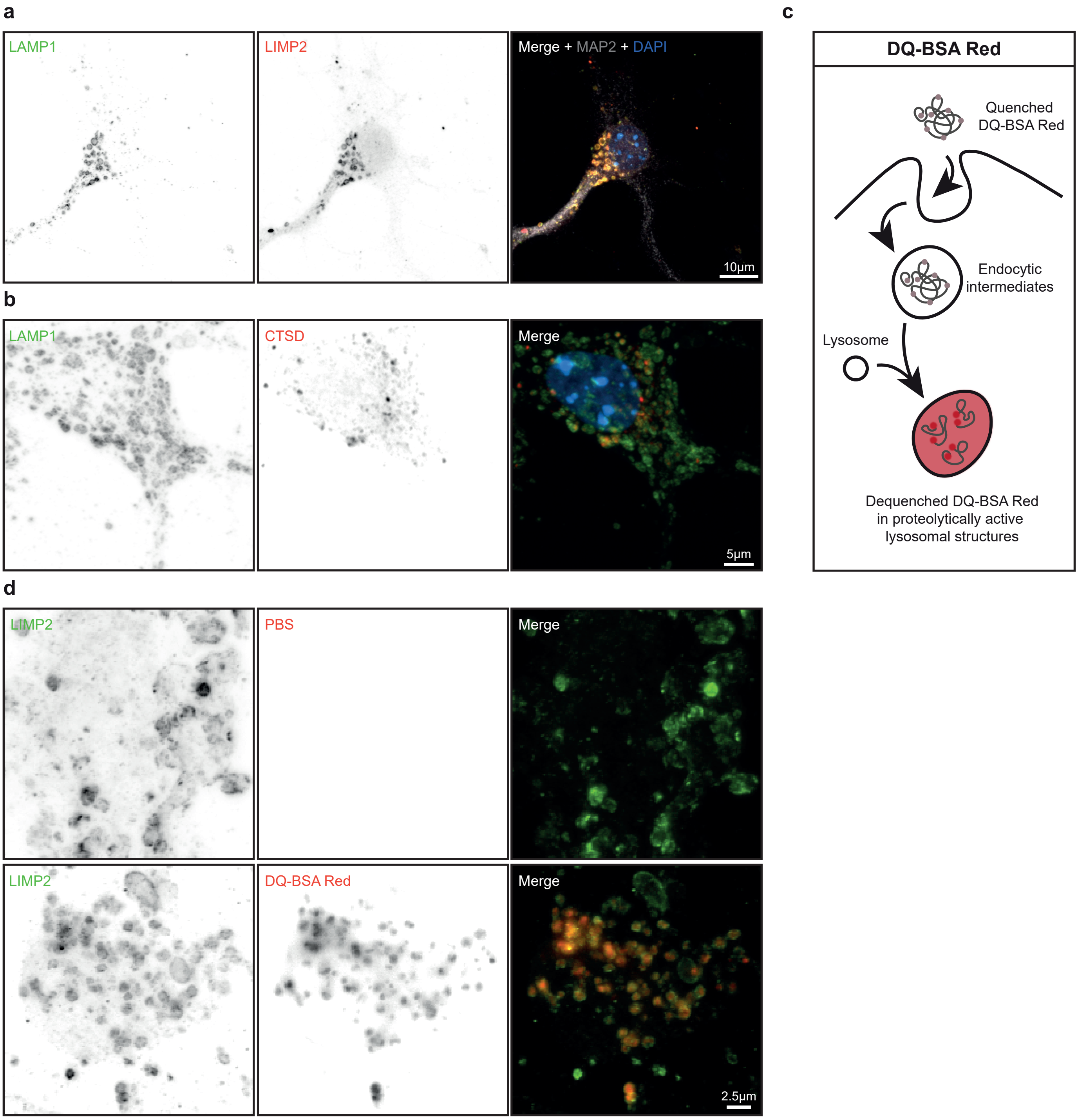


**Supplementary Fig. 12** Localization of the lysosomal markers used in this study. **a** Primary cortical mouse neurons (DIV 8) immunolabeled using antibodies against the transmembrane proteins LAMP1 and LIMP2 marking the late compartments of the autophagic and endocytic pathways (shown separately in grayscale and in color in the merge: LAMP1 in green and LIMP2 in red). **b** Primary hippocampal mouse neurons (DIV 18, PFF treated) immunolabeled using antibodies against LAMP1 and the lysosomal hydrolase CTSD (shown separately in grayscale and in color in the merge: LAMP1 in green and CTSD in red). Cell nuclei are stained with DAPI (blue) in **a** and **b**. **c** Schematic representation of the mechanism of the DQ-BSA assay. Self-quenched DQ-BSA is endocytosed in live cells and dequenched upon hydrolysis in proteolytically active compartments. **d** Primary hippocampal mouse neurons (DIV 18) treated for 18 hours with DQ-BSA to visualize sites of proteolysis or with PBS as a control and immunolabeled with LIMP2 (shown separately in grayscale and in color in the merge: LIMP2 in green and PBS/DQ-BSA in red)

# Supplementary Figure 13

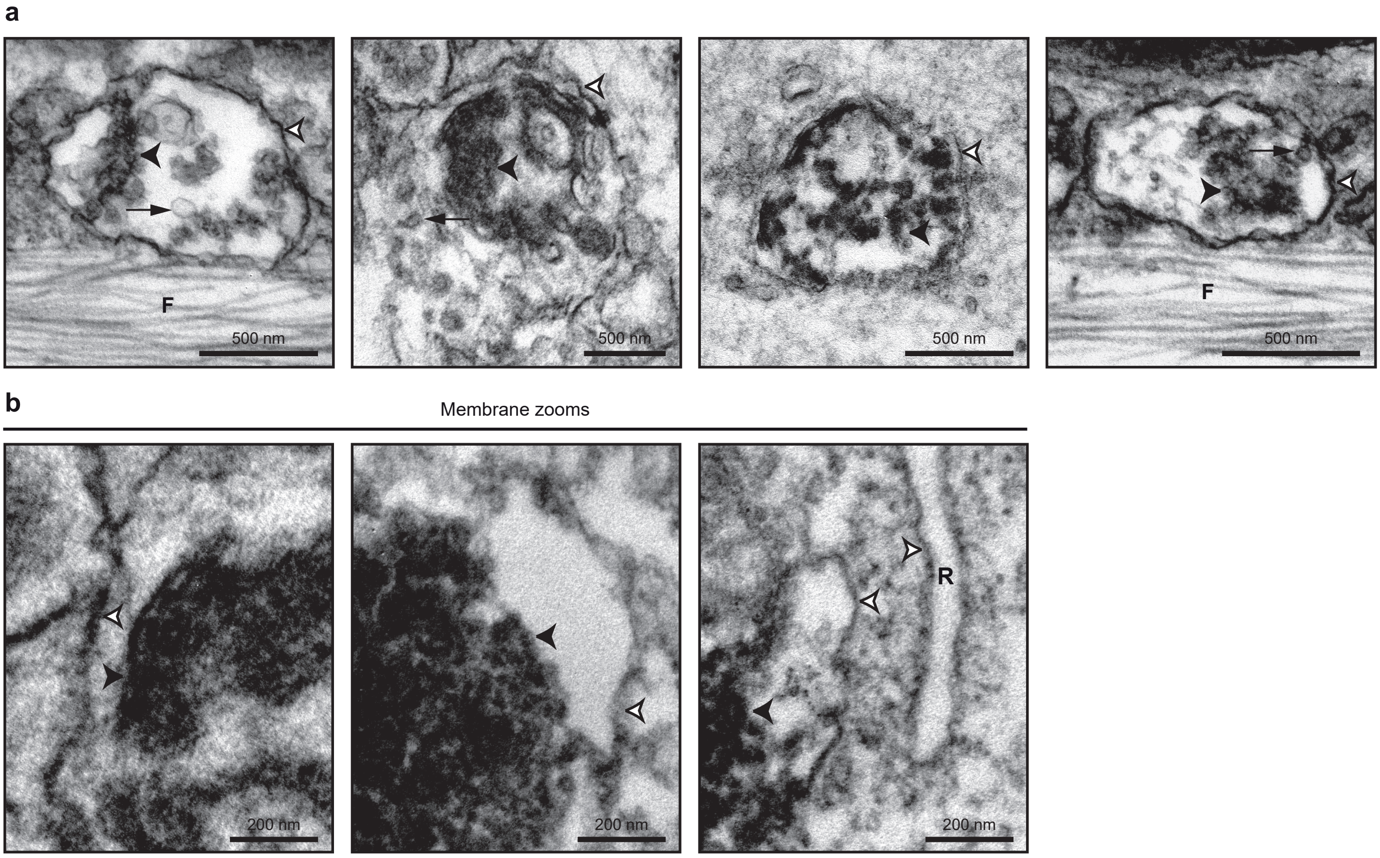


**Supplementary Fig. 13** CK1δ-positive GVBs are delineated by a single membrane. Primary mouse neurons expressing tau P301L were treated with PFFs and GVBs were identified by CK1δ-DAB immunocytochemistry and electron microscopy. **a** Electron micrographs showing GVBs with one or multiple CK1δ-positive core(s), a clear vacuole frequently containing small vesicular structures. Both cores and vacuole were enclosed by a single limiting membrane. **b** Micrographs of membrane zooms of single membrane structures, GVBs and endoplasmic reticulum (R), are shown. In micrographs, fibrillar structures (F), CK1δ-positive cores (black arrowheads), small vesicular structures (small black arrows) and single membranes (single white arrowheads with black border) are indicated

# Supplementary Figure 14

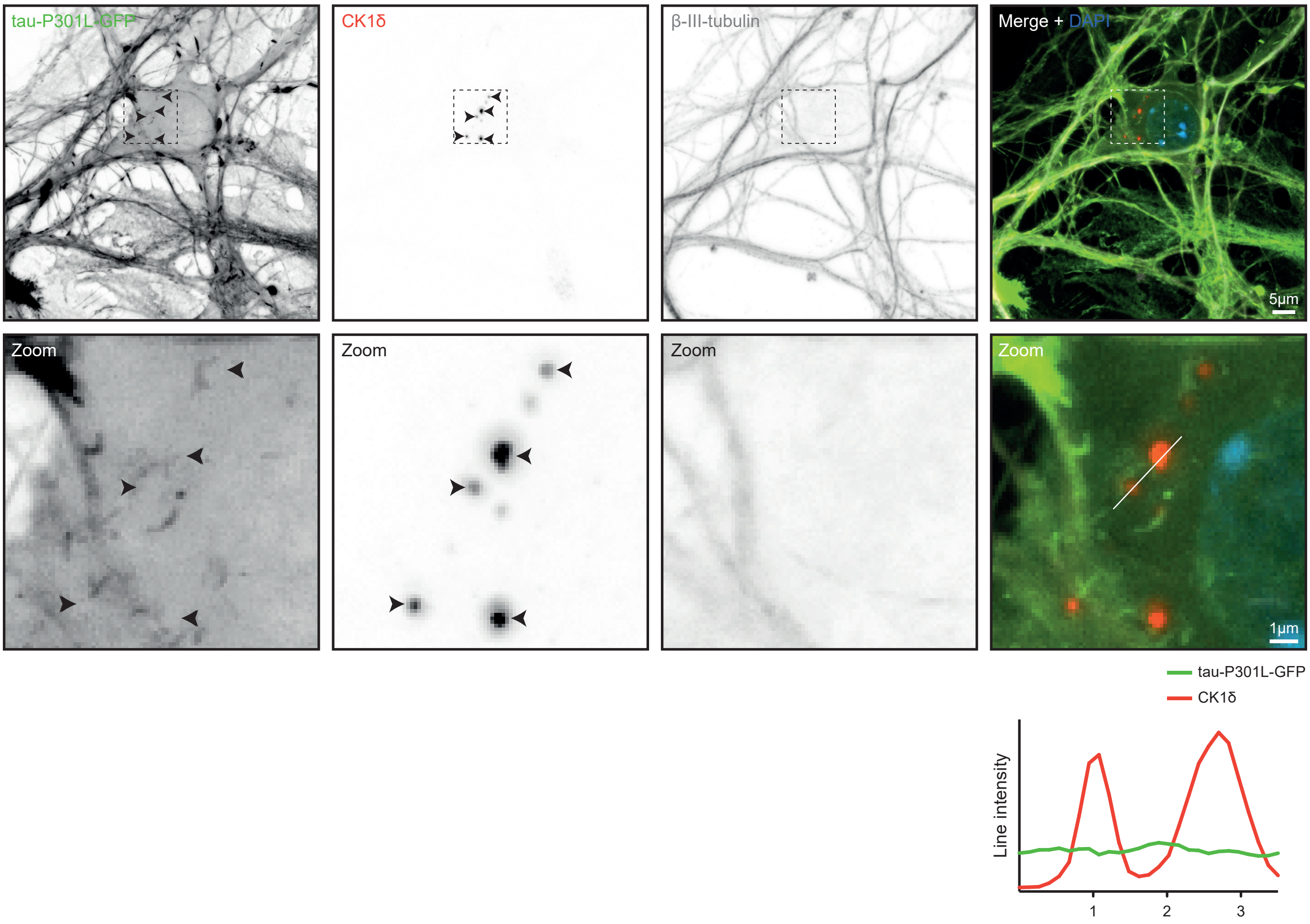


**Supplementary Fig. 14** Non-aggregated tau does not accumulate in the core of GVBs. Tau pathology was induced in primary mouse neurons expressing tau-P301L-GFP by treatment with PFFs. GVB-positive neurons without abundant somatic tau pathology were selected for analysis to enable analysis of the lower signal of diffusely distributed tau in the cytosol and GVBs without interference of the strong fluorescent signal from tau aggregates. Representative confocal images, zooms and fluorescence intensity profile of direct fluorescence signal from tau-P301L-GFP and immunostaining using the GVB core marker CK1δ and neuron-specific microtubule marker β-III-tubulin are shown. Individual fluorescence signals are shown separately in grayscale and in color in the merge: tau-P301L-GFP in green, CK1δ in red and β-III-tubulin in gray. Cell nuclei are stained with DAPI (blue). Zoomed areas are indicated by dashed boxes. Arrowheads point to examples of GVBs. Intensity profiles are determined along the line segment shown as a white bar in the merge of the zooms. See Supplementary Table 3 in Online Resource 1 for an overview of the number of cells analyzed

# Supplementary Figure 15

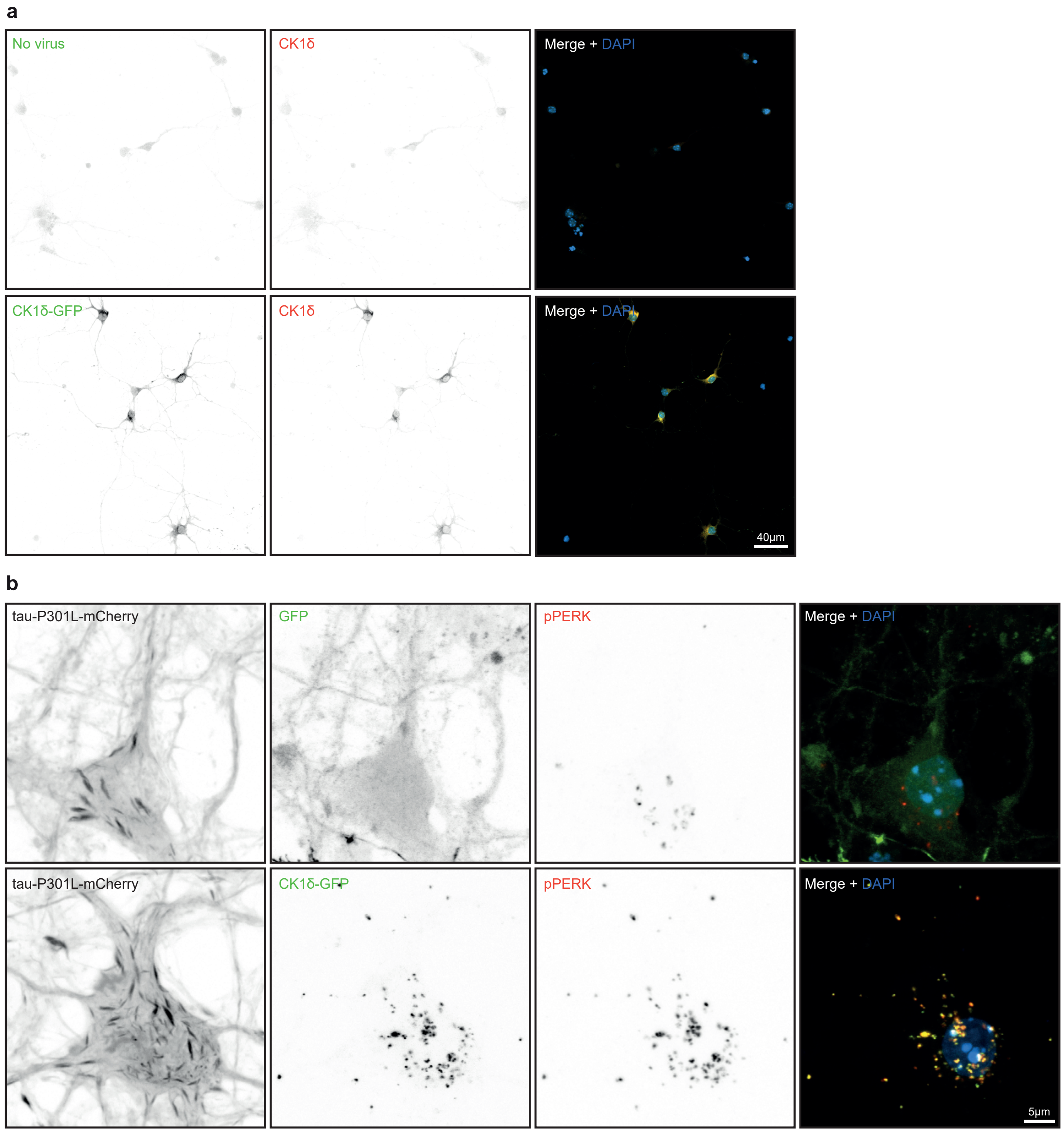


**Supplementary Fig. 15** CK1δ is specifically targeted to GVBs. **a** Representative confocal images of primary hippocampal mouse neurons with or without lentiviral-mediated expression of CK1δ-GFP immunolabeled with CK1δ. Individual fluorescence signals are shown separately in grayscale and in color in the merge: CK1δ-GFP in green and CK1δ immunoreactivity in red. **b** Representative confocal images of neurons expressing tau-P301L-mCherry showing the expression of either GFP or CK1δ-GFP combined with immunolabelling for the GVB marker pPERK. Individual fluorescence signals are shown separately in grayscale and in color in the merge: GFP and CK1δ-GFP in green and pPERK in red. Tau-P301L-mCherry signal is not shown in the merge. See Supplementary Table 3 in Online Resource 1 for an overview of the number of cells analyzed. Cell nuclei are stained with DAPI (blue) in all images

**Supplementary Table 1** Overview of the human cases included in the present study.

| Source | Application | Neuropatholo-gical diagnosis | Sex | Age at death | Disease duration | Cause of death | PMI (hrs:min) | NFT stage | Amyloid stage | Other pathologies | Brain region used |
|---|---|---|---|---|---|---|---|---|---|---|---|
| Netherlands brain bank | IF | AD | F | 66 | 3 | Euthanasia * | 08:15 | 5 | C | Atypical/anterior α-synucleinopathy | Hippocampus |
| Netherlands brain bank | IF | AD | M | 70 | 9 | Respiratory insufficiency | 04:50 | 6 | C | - | Hippocampus |
| Netherlands brain bank | IHC | AD | F | 67 | 9 | Pneumonia | 06:05 | 6 | C | - | Hippocampus |
| University Hospital Basel | Homogenate for injection | Ctrl | M | 76 | n.a. | Pneumonia | 18:00 | 1 | A | Moderate cribriform state in both striata, one lacuna in white matter of right parietal lobe | Temporal cortex |
| Choju Medical Institute | Homogenate for injection | AD (AD - 1) | F | 89 | 2 | Pneumonia | 08:00 | 5 | C | Multiple old infarcts | Temporal cortex |
| Choju Medical Institute | Homogenate for injection | AD (AD – 2) | F | 86 | 12 | Heart failure | 13:00 | 6 | C | Single old infarct in left temporal lobe, middle cerebral artery occlusion | Temporal cortex |
| Choju Medical Institute | Homogenate for injection | PSP | M | 68 | 10 | Multiple organ failure | 54:00 | 2 | A | - | Putamen |
| Choju Medical Institute | Homogenate for injection | TD | F | 85 | 15 | Heart failure | 09:00 | 5/6 | A | Embolism | Hippocampus |

Source and application of the tissue are listed. Neuropathological diagnosis, sex, age at death and disease duration in years, cause of death, post-mortem interval in hours to minutes, neurofibrillary tangle stage and amyloid stage according to [2], other pathologies and brain region used in the present study are listed per case. Homogenates used for injection of mice were analyzed by Western blotting for the presence of Aβ [3] and tau (Supplementary Fig. 2a in Online Resource 1). * in accordance with Dutch law # BWBR0012410. AD = Alzheimer's disease (AD-1 and AD-2 refer to Western blots in Supplementary Fig. 2a in Online Resource 1), Ctrl = non-neurological control case, F = female, hrs:min = hours to minutes, IF = immunofluorescence, IHC = immunohistochemistry, M = male, n.a.= not applicable, NFT = neurofibrillary tangle, PMI = post-mortem interval, PSP = progressive supranuclear palsy, TD = tangle-only dementia, - = not detected

**Supplementary Table 2** Overview of the number of mice, sections and cells analyzed in the *in vivo* recombinant tau seeding model.

| Figure | Number of mice analyzed | | Number of sections analyzed per mouse | Number of cells analyzed | |
|---|---|---|---|---|---|
| | **Ctrl** | **PFF** | | **Ctrl** | **PFF** |
| Fig. 1c | 5 | 5 | 2 | 3182 | 2457 |
| Fig. 1d | 5 | 5 | 1 | 1463 | 1266 |
| Fig. 1e | - | 5 | 1 | - | 181 |
| Fig. 1f | - | 5 | 1 | - | 90 |
| Supplementary Fig. 1b | 5 | 4 | 1 | 1719 | 1191 |
| Supplementary Fig. 1c | - | 4 | 1 | - | 154 |
| Supplementary Fig. 1d | - | 4 | 1 | - | 62 |

(Supplementary) figure number, number of analyzed control buffer- (Ctrl) and PFF-injected animals, number of sections analyzed per mouse and total number of cells analyzed are shown

**Supplementary Table 3** Overview of the number of independent experiments and analyzed cells in primary mouse neurons with PFF-seeded tau pathology processed for immunofluorescence to visualize GVBs.

| **Figure** | **Experimental details** | **Number of independent experiments** | **Number of cells analyzed** |
|---|---|---|---|
| Fig. 2a | CK1δ | >4 | >100 |
| Fig. 2b | CK1δ + pPERK | 3 | 31 |
| Fig. 2c | CK1δ + peIF2α | 3 | 26 |
| Fig. 2d | CK1δ + pIRE1α | 3 | 16 |
| Fig. 2e | CK1δ + CK1ε | 4 | 32 |
| Fig. 2f | CK1δ + CHMP2B | 3 | 45 |
| Fig. 3d | CK1δ | >4 | >100 |
| Fig. 5a | CK1δ + LC3 | 3 | 35 |
| Fig. 5b | CK1δ + EEA1 | 3 | 28 |
| Fig. 5c | CK1δ + LIMP2 | 3 | 53 |
| Fig. 5d | CK1δ + CHMP2B + LAMP1 | 3 | 59 |
| Fig. 6a | CK1δ + CTSD + LAMP1 | 3 | 47 |
| Fig. 8a | CK1δ + AT100 | 3 | 38 |
| Fig. 8b | CK1δ + tau-P301L-GFP | >4 | >100 |
| Fig. 8c | CK1δ + tau-P301L-GFP | 3 | 137 |
| Fig. 8d | CK1δ + Myc | 3 | 21 |
| Fig. 8g | CK1δ-GFP + LIMP2 | 3 | 30 |
| Supplementary Fig. 11a | CK1δ + GRASP55 | 3 | 29 |
| Supplementary Fig. 11b | CK1δ + Calnexin | 3 | 26 |
| Supplementary Fig. 11c | CK1δ + VGLUT1 | 3 | 31 |
| Supplementary Fig. 11d | CK1δ + VGAT | 3 | 40 |
| Supplementary Fig. 11e | CK1δ + G3BP | 3 | 55 |
| Supplementary Fig. 14 | CK1δ + tau-P301L-GFP | 3 | 28 |
| Supplementary Fig. 15b | GFP + pPERK | 2 | 13 |
| Supplementary Fig. 15b | CK1δ-GFP + pPERK | 2 | 35 |

(Supplementary) figure number, details of the experiments (immunostainings/lentiviral constructs), number of independent experiments and analyzed cells are listed

**References accompanying Supplementary Figures and Supplementary Tables**


1. Clavaguera F, Bolmont T, Crowther RA, Abramowski D, Frank S, Probst A, Fraser G, Stalder AK, Beibel M, Staufenbiel M, Jucker M, Goedert M, Tolnay M (2009) Transmission and spreading of tauopathy in transgenic mouse brain. Nat Cell Biol 11:909–913. doi: 10.1038/ncb1901
2. Braak H, Braak E (1991) Neuropathological stageing of Alzheimer-related changes. Acta Neuropathol 82:239–259. doi: 10.1007/BF00308809
3. Clavaguera F, Akatsu H, Fraser G, Crowther RA, Frank S, Hench J, Probst A, Winkler DT, Reichwald J, Staufenbiel M, Ghetti B, Goedert M, Tolnay M (2013) Brain homogenates from human tauopathies induce tau inclusions in mouse brain. Proc Natl Acad Sci 110:9535–9540. doi: 10.1073/pnas.1301175110
